# A cannabidiol aminoquinone derivative activates the PP2A/B55α/HIF pathway and shows protective effects in a murine model of traumatic brain injury

**DOI:** 10.1186/s12974-022-02540-9

**Published:** 2022-07-09

**Authors:** Carmen Navarrete, Adela García-Martín, Alejandro Correa-Sáez, María E. Prados, Francisco Fernández, Rafael Pineda, Massimiliano Mazzone, Marina Álvarez-Benito, Marco A. Calzado, Eduardo Muñoz

**Affiliations:** 1 Emerald Health Pharmaceuticals, San Diego, USA; 2 Maimonides Biomedical Research Institute of Córdoba, University of Córdoba, Avda Menéndez Pidal s/n, 14004 Córdoba, Spain; 3 Cellular Biology, Physiology and Immunology Department, University of Cordoba, Córdoba, Spain; 4 Hospital Universitario Reina Sofía, Córdoba, Spain; 5 VivaCell Biotechnology España, Córdoba, Spain; 6 FEA Radiodiagnóstico, Sección de Neurorradiología Diagnóstica. Hospital Universitario Reina Sofía, Córdoba, Spain; 7 Laboratory of Tumor Inflammation and Angiogenesis, Center for Cancer Biology, VIB-KULeuven, 3000 Louvain, Belgium; 8 Unidad de Radiodiagnóstico Y Cáncer de Mama, Hospital Universitario Reina Sofía, Córdoba, Spain

**Keywords:** Traumatic brain injury, Protein phosphatase 2A, Hypoxia-inducible factor, Prolyl-hydroxylases, Brain–blood barrier, Neuroprotection

## Abstract

**Background:**

Traumatic brain injury (TBI) is characterized by a primary mechanical injury and a secondary injury associated with neuroinflammation, blood–brain barrier (BBB) disruption and neurodegeneration. We have developed a novel cannabidiol aminoquinone derivative, VCE-004.8, which is a dual PPARγ/CB_2_ agonist that also activates the hypoxia inducible factor (HIF) pathway. VCE-004.8 shows potent antifibrotic, anti-inflammatory and neuroprotective activities and it is now in Phase II clinical trials for systemic sclerosis and multiple sclerosis. Herein, we investigated the mechanism of action of VCE-004.8 in the HIF pathway and explored its efficacy in a preclinical model of TBI.

**Methods:**

Using a phosphoproteomic approach, we investigated the effects of VCE-004.8 on prolyl hydroxylase domain-containing protein 2 (PHD2) posttranslational modifications. The potential role of PP2A/B55α in HIF activation was analyzed using siRNA for B55α. To evaluate the angiogenic response to the treatment with VCE-004.8 we performed a Matrigel plug in vivo assay. Transendothelial electrical resistance (TEER) as well as vascular cell adhesion molecule 1 (VCAM), and zonula occludens 1 (ZO-1) tight junction protein expression were studied in brain microvascular endothelial cells. The efficacy of VCE-004.8 in vivo was evaluated in a controlled cortical impact (CCI) murine model of TBI.

**Results:**

Herein we provide evidence that VCE-004.8 inhibits PHD2 Ser125 phosphorylation and activates HIF through a PP2A/B55α pathway. VCE-004.8 induces angiogenesis in vivo increasing the formation of functional vessel (CD31/α-SMA) and prevents in vitro blood–brain barrier (BBB) disruption ameliorating the loss of ZO-1 expression under proinflammatory conditions. In CCI model VCE-004.8 treatment ameliorates early motor deficits after TBI and attenuates cerebral edema preserving BBB integrity. Histopathological analysis revealed that VCE-004.8 treatment induces neovascularization in pericontusional area and prevented immune cell infiltration to the brain parenchyma. In addition, VCE-004.8 attenuates neuroinflammation and reduces neuronal death and apoptosis in the damaged area.

**Conclusions:**

This study provides new insight about the mechanism of action of VCE-004.8 regulating the PP2A/B55α/PHD2/HIF pathway. Furthermore, we show the potential efficacy for TBI treatment by preventing BBB disruption, enhancing angiogenesis, and ameliorating neuroinflammation and neurodegeneration after brain injury.

**Supplementary Information:**

The online version contains supplementary material available at 10.1186/s12974-022-02540-9.

## Introduction

Traumatic brain injury (TBI) is one of the most severe kinds of injury related with both cause of death and long-term implications for survivors. Currently it is the leading cause of injury-related death and disability in the United States with a very similar incidence in Europe [1, 2]. The pathophysiology of TBI involves several types of damage, usually classified as primary and secondary insults, making it a complex pathology to address. TBI is induced by the application of mechanical force to the head, for instance, brain contusion, and stretching of the brain tissue produced by movement of the brain structures relative to the skull. These alterations trigger a series of secondary injuries that could be manifested over a period of hours, days and even months depending in part on the nature and location of the injury. TBI promotes disruption of brain–blood barrier (BBB) integrity causing edema, vascular leakage, hemorrhage and hypoxia, and exposure of peripheral components to the brain parenchyma [3, 4]. BBB does not work individually, but it is part of the larger complex of a multicellular neurovascular unit (NVU) that includes neurons, astrocytes, pericytes, microglia and the blood vessels [5]. In fact, the endothelial cells which compose the brain blood vessels are the anatomical substrate of the BBB and the NVU controlling the transcellular and paracellular transport mechanism [6, 7]. TBI can initiate cerebrovascular pathology by first damaging endothelial cells and consequently triggering BBB dysfunction, increasing the activation of microglia and astrocytes, enhancing the inflammatory response [8].

Hypoxia-inducible factors (HIFs) are transcription factors controlled by a cellular low oxygen status. They modulate several biological activities such as angiogenesis, vascular tone and immunity through regulating the expression of genes related with these processes. The stability of HIF-1α is modulated by the HIF prolyl hydroxylases (PHDs) that under normal oxygenic conditions hydroxylate HIF-1α leading to its ubiquitination and degradation by the proteosome. HIF-1α activation induced by enzymatic inhibitors of PHDs (PHDi) is of translational interest for the treatment of ischemic and inflammatory conditions [9]. Furthermore, this class of PHDi is either in Phase III clinical trials or approved in some countries for the treatment of anemia in chronic kidney disease patients [10, 11].

More recently, it has been shown that a second level of regulation of PHD2 activation is mediated by B55α, a subunit of the protein phosphatase 2A (PP2A) complex [12]. B55α induces the post-transcriptional modification of PHD2 at Ser125 that is closely associated with the stabilization of HIF-1α protein [12]. Importantly, PP2A/B55α is the brain primary Tau phosphatase that dephosphorylates Tau protein and its deficiency may play an important role in tauopathies [13]. Traumatic brain injury (TBI) is an important risk factor for neurodegeneration and dementia, such as chronic traumatic encephalopathy (CTE). The primary mechanisms that have much in common in the neuropathology in TBI and CTE patients involve hyperphosphorylated Tau pathology, axonal degeneration, neuronal loss and seizure [14–16]. In this regard, PP2A/B55α expression is reduced in a rat model of repeated mild TBI and restoring its expression with sodium selenite it has been shown to exert neuroprotective effects mitigating hyperphosphorylation of tau [17]. In addition, and of special importance for neuroinflammatory diseases including TBI where the BBB is compromised, novel activities related with endothelial protection and vascular remodeling have been demonstrated for the PP2A/B55α isoform [18].

VCE-004.8 is a cannabidiol aminoquinone that is a dual PPARγ/CB_2_ agonist that also activates the HIF pathway [19, 20], and we show here for the first time that PP2A/B55α is the main target of VCE-004.8 for HIF activation. Partially due to this novel activity on PP2A/B55α that is involved in vascular remodeling, and combined with its anti-inflammatory effects [19], VCE-004.8 can be considered a potentially effective molecule for the treatment of TBI.

## Methods

### In vitro experiments

#### Cell lines and reagents

HEK-293T (ATCC® CRL-3216™) and NIH-3T3-EPO-Luc cells were maintained at 37 °C in a humidified atmosphere containing 5% CO_2_ in DMEM supplemented with 10% fetal bovine serum (FBS), 2 mM l-glutamine, and 1% (v/v) penicillin/streptomycin. Human microvascular endothelial cells (HMEC-1; ATCC® CRL-3243™) were maintained in MCDB131 medium (Life Technologies) supplemented with 10 ng/mL epidermal growth factor (EGF), 1 μg/mL hydrocortisone, 10 mM glutamine and 10% FBS. The mouse NIH-3T3-EPO-Luc cells have been stably transfected with the Epo-Luc plasmid that contains three copies of the hypoxia response element (HRE) sequence from the promoter of the erythropoietin gene in the pGL3 vector. Immortalized mouse brain microvascular endothelial cell line bEnd5 (it was kindly gifted by Dr. Carmen Guaza, Cajal Institute, Madrid, Spain) was cultured in complete medium containing DMEM supplemented with 2 mM glutamine, 1 mM sodium pyruvate, 1% non-essential amino acids, 10% FBS and 1% penicillin/streptomycin. All other reagents were purchased from Sigma (St Louis, MO, USA). HA-PHD1, HA- PHD2, HA-PHD3 and GST-PHD plasmids were described previously [21]. Scramble control oligonucleotide siRNA non-targeting pool (#D-001810) and ON-TARGET plus SMARTpool against B55α (#L-004824) were purchased from Dharmacon (Waltham, MA, USA).

#### Western blots

Western blot was performed as previously described [19]. Briefly, after treatments, the cells were washed with PBS and proteins extracted in 50 μL of lysis buffer (50 mM Tris–HCl (pH 7.5), 150 mM NaCl, 10% glycerol, and 1% NP-40) supplemented with 10 mM NaF, 1 mM Na3VO4, 10 μg/mL leupeptin, 1 μg/mL pepstatin and aprotinin, and 1 μL/mL PMSF saturated. Thirty micrograms of proteins were boiled at 95 °C in Laemmli buffer and electrophoresed in 10% SDS/PAGE gels. Immunodetection of specific proteins was carried out by incubation with primary antibody against HIF-1α (1:1000 dilution, #610,959, BD Biosciences, San Jose, CA, USA), OH-HIF-1α (1:1000 dilution, #3434S, Cell Signaling, Danvers, MA, USA), B55α (1:1000 dilution, #5689S, Cell Signaling), anti-HA (1:1000 dilution, #clone 3F10 Roche), anti-Phospho-PHD2 Ser125 (1:500 dilution) [10], anti-PHD1 (1:1000 dilution, #ab108980, Abcam), anti-PHD2 (1:1000 dilution, #ab109088, Abcam), PHD3 (1:1000 dilution, #ab30782, Abcam) and β-actin (1:10.000 dilution, #ab49900, Abcam, Cambridge, UK), overnight at 4 °C. After washing membranes, horseradish peroxidase-conjugated secondary antibody was added and detected by chemiluminescence system (GE Healthcare Europe GmbH).

### Determination of PHD2 posttranslational modifications

#### Cell transfections and immunoprecipitations

B55α silencing was performed with Lipofectamine RNAiMax transfection reagent (#13,778,100, Life Technologies, Carlsbad, USA) according to the manufacturer’s instructions. Transient transfections with pcDNA3-HA-PHD2 were performed in HEK-293T cells with Roti-Fect (Carl Roth, Karlsruhe, Germany). After 48 h the cells were collected, washed in PBS and lysed in immunoprecipitation (IP) buffer (50 mM Hepes pH 7.5, 50 mM NaCl, and 1% Triton X-100) supplemented with 5 mM EGTA, 20 mM Na4P2O7, 50 mM NaF, 1 mM Na3VO4, 2 mM PMSF, and 10 μg/mL of leupeptin, aprotinin and pepstatin. Immunoprecipitation was performed with 1 μg of the indicated antibodies and 25 μL of protein A/G Sepharose beads. Immunoprecipitated proteins were then five times washed in IP buffer and eluted in 2 × SDS sample buffer, followed by Western blotting.

#### Sample preparation for LC–MS/MS

Beads used in immunoprecipitation were cleaned three times with 500 µL of 200 mM ammonium bicarbonate and 60 µL of 6 M Urea/200 mM ammonium bicarbonate were added. Samples were then reduced with dithiothreitol (30 nmol) (Sigma) at 37 °C for 60 min, alkylated in the dark with iodoacetamide (60 nmol, 25 °C, 30 min) and diluted to 1 M urea with 200 mM ammonium bicarbonate for trypsin digestion (1 µg) (cat # V5113, Promega, Madison, WI, USA) for 8 h at 37 ºC. After digestion, the peptide mix was acidified with formic acid and desalted with a MicroSpin C18 column (The Nest Group, Inc) prior to LC–MS/MS analysis.

#### Chromatographic and mass spectrometric analysis

Samples were analyzed using an LTQ-Orbitrap Fusion Lumos mass spectrometer (Thermo Fisher Scientific, San Jose, CA, USA) coupled to an EASY-nLC 1000 (Thermo Fisher Scientific (Proxeon), Odense, Denmark). Peptides were loaded directly onto the analytical column and were separated by reversed-phase chromatography using a 50-cm column with an inner diameter of 75 μm, packed with 2 μm C18 particles spectrometer (Thermo Scientific). Chromatographic gradients started at 95% buffer A and 5% buffer B with a flow rate of 300 nL/min for 5 min and gradually increased to 25% buffer B and 75% A in 52 min and then to 40% buffer B and 60% A in 8 min. After each analysis, the column was washed for 10 min with 10% buffer A and 90% buffer B (buffer A: 0.1% formic acid in water; buffer B: 0.1% formic acid in 80% acetonitrile. The mass spectrometer was operated in positive ionization mode with nanospray voltage set at 2.4 kV and source temperature at 275 °C. Ultramark 1621 was used for external calibration of the FT mass analyzer prior the analyses, and an internal calibration was performed using the background polysiloxane ion signal at m/z 445.1200. The acquisition was performed in data-dependent acquisition (DDA) mode and full MS scans with 1 micro scan at a resolution of 120,000 were used over a mass range of m/z 350–1500 with detection in the Orbitrap mass analyzer. Auto gain control (AGC) was set to 1E5 and charge state filtering disqualifying singly charged peptides was activated. In each cycle of data-dependent acquisition analysis, following each survey scan, the most intense ions above a threshold ion count of 10,000 were selected for fragmentation. The number of selected precursor ions for fragmentation was determined by the “Top Speed” acquisition algorithm and a dynamic exclusion of 60 s. Fragment ion spectra were produced via high-energy collision dissociation (HCD) at normalized collision energy of 28% and they were acquired in the ion trap mass analyzer. AGC was set to 1E4, and an isolation window of 1.6 m/z and a maximum injection time of 200 ms were used. Digested bovine serum albumin (# P8108S, New England Biolabs, Ipswich, MA, USA) was analyzed between each sample to avoid sample carryover and to assure stability of the instrument; QCloud was used to control instrument longitudinal performance.

#### LC–MS/MS data analysis

Acquired spectra were analyzed using the Proteome Discoverer software suite (v1.4, Thermo Fisher Scientific) and the Mascot search engine (v2.6, Matrix Science). The data were searched against a Swiss-Prot human database plus a list of common contaminants and all the corresponding decoy entries. For peptide identification a precursor ion mass tolerance of 7 ppm was used for MS1 level; trypsin was chosen as enzyme and up to three missed cleavages were allowed. The fragment ion mass tolerance was set to 0.5 Da for MS2 spectra. Phosphorylation of serine, threonine and tyrosine, oxidation of methionine and N-terminal protein acetylation were used as variable modifications whereas carbamide methylation on cysteines was set as a fixed modification. Precursor area of phosphorylated peptides were extracted with the Skyline-daily software (v20.1.1.83), median of the area for each condition and fold change was calculated. False discovery rate (FDR) in peptide identification was set to a maximum of 5%. SAINT express algorithm was used to score protein–protein interactions.

#### Luciferase assays

For EPO-Luc transactivation as a marker of HIF stabilization, NIH-3T3-EPO-luc cells were seeded in 96-well plates and incubated with the pan-PP2A inhibitors LB-100 (#S7537, Selleckchem, Houston, USA) and okadaic acid sodium salt (#459,620 Merck) as indicated for 30 min before VCE-004.8 compound was added. Luciferase activity was quantified using Dual-Luciferase Assay (#E1483, Promega, Madison, WI, USA) after 6 h of stimulation.

#### Quantitative reverse transcriptase-PCR analyses

Total RNA was isolated from pericontusional brain area using QIAzol lysis reagent and the Rneasy Lipid mini kit (#74,804, Qiagen, Hilden, Germany) according to the manufacturer’s instructions. For quantitative reverse transcriptase-PCR assays, total RNA (1 μg) was retrotranscribed using the iScript cDNA Synthesis Kit (#1,708,891, Bio-Rad, Hercules, CA, USA) and the cDNA was analyzed by real-time PCR using the iQTM SYBR Green Supermix (#1,708,880 Bio-Rad) and a CFX96 Real-time PCR Detection System (Bio-Rad). Cyclophilin A (Ppia) gene was used to standardize mRNA expression in each sample. Gene expression was quantified using the 2^− ΔΔCt^ method, and the percentage of relative expression against sham animals was represented. The primers used in this study were acquired in Eurofins (Luxembourg) and are listed in the following table:

**Table:** 

Genes	Forward (5′–3′)	Reverse (5′–3′)
Il-6	GTATGAACAACGATGATGCACTTG	ATGGTACTCCAGAAGACCAGAGGA
Il-1β	CTCCACCTCAATGGACAGAA	GCCGTCTTTCATTACACAGG
Ccl2	GGGCCTGCTGTTCACAGTT	CCAGCCTACTCATTGGGAT
Il-10	GGTTGCCAAGCCTTATCGGA	ACCTGCTCCACTGCCTTGCT
Ppia	CATACAGGTCCTGGCATCTTGTC	AGACCACATGCTTGGCATCCAG

#### Transendothelial electrical resistance (TEER) in brain microvascular endothelial cells

Mouse brain microvascular endothelial cells bEnd5 were seeded at a density of 20,000 cells/cm^2^ on the 24-well on polycarbonates inserts (6.5 mm diameter, 0.4 μm pore size) (#CLS3413–48EA, Sigma) and every other day growth medium was renewed. On day 7 after seeding, apical growth medium was supplemented with 100 nM hydrocortisone (HC) (100 μM stock dissolved in ethanol). Every 2 days, the apical medium was exchanged containing 100 nM HC. On day 12, apical medium was exchanged with HC and treated with VCE-004.8 (5 µM) for 24 h. On day 13, treatment with 200 μL of human peripheral blood mononuclear cell (PBMC) supernatant previously activated with phytohemagglutinin-M (PHA) for 48 h was added for 30 min. Transendothelial electrical resistance (TEER) was measured using a Millicell® ERS-2 (Electrical Resistance System, Millipore, MA, USA) in each well (vol/vol). The TEER values on bEnd5 cells were calculated by subtracting the blank resistance (background electrical resistance from an insert without cells including filter and medium) and multiplying that result by the effective growth area of the membrane (0.6 cm^2^). The results are expressed as a percentage of endothelial disruption, taking 0% as the disruption in untreated cells. Inserts were cryopreserved and immunofluorescence studies were performed to determine the expression of claudin-5 (CLDN5) and zonula occludens 1 (ZO-1) by confocal microscopy.

#### Immunocytochemistry analysis

bEnd5 cells (2.5 × 10^3^/well) were seeded onto glass coverslips in 24‐well plates. Cells were pre‐stimulated with VCE‐004.8 at several concentrations for 1 h and incubated for 24 h with interleukin 1 beta (IL1β) (R&D Systems, Minneapolis, MN, USA) plus tumor necrosis factor alpha (TNFα) (R&D Systems) (for VCAM1 study) or interleukin 6 (IL-6) (R&D Systems) plus TNFα (for ZO-1 study). Cells were then washed with PBS and fixed with 4% formaldehyde for 10 min at room temperature (RT). After fixation, cells were washed twice with PBS, permeabilized with 0.5% Triton X-100 in PBS at RT for 5 min and blocked in PBS with 3% BSA for 1 h. Cells were incubated overnight at 4 °C with the following primary antibodies diluted in PBS with 3% BSA: rabbit monoclonal anti-VCAM1 (1:100, #ab134047, Abcam) or rabbit polyclonal anti-ZO-1 (1:100, #10,222,233, Invitrogen; Carlsbad, CA, USA). Next, cells were washed three times with PBS and incubated with the secondary antibody anti-rabbit Texas Red (1:100, #A-6399, Thermo Fischer Scientific) for 1 h at RT. Finally, coverslips were mounted with Vectashield Mounting Medium with 4′,6-diamidino-2-phenylindole (DAPI) (Vector Laboratories, Burlingame, CA, USA) for nuclear staining. Images were acquired using a spectral confocal laser-scanning microscope LSM710 (Zeiss, Jena, Germany) with 25 × /0.8 Plan-Apochromat oil immersion lens.

### In vivo experiments

#### Animals

The animal model was performed in strict accordance with the European Union (EU) and governmental regulations. Handling of animals was performed in compliance with the guidelines of animal care set by the EU guidelines 86/609/EEC, and the Ethic Committee on Animal Experimentation at the University of Córdoba (UCO, Córdoba, Spain) approved all the procedures described in this study (numbers 2020PI/14 and 2018PI/03 for TBI for angiogenesis in vivo, respectively). Briefly, all animals were housed in the animal facilities under the following controlled conditions: 12 h light/dark cycle; temperature 20 °C (± 2 °C) and 40–50% relative humidity with free access to standard food and water.

#### Controlled cortical impact (CCI) injury model

CCI is a commonly used and highly regarded model of brain trauma to induce reproducible and well-controlled injury [22]. A moderate TBI model was established in C57BL/6 male mice at 12–14 weeks of age (*n* = 10 animals per group) using an electronic cortical precise impactor (#68099II RWD, 6540 Lusk Blvd, Suite C161 San Diego, CA 92,121 USA). For inducing the CCI model, mice were intraperitoneally anesthetized using xylazine (10 mg/kg)/ketamine (125 mg/kg) and then placed on a stereotaxic apparatus. The coronal and sagittal sutures were fully exposed, and a craniotomy (5 mm diameter) was performed midway between bregma and lambda with the medial edge 1 mm lateral to the midline leaving the dura intact. Mice were impacted at ≈ 4.5 m/s with a 0.2 s dwell time and 2 mm depth using a 3-mm-diameter impact tip. The scalp incision was closed using a surgical adhesive. Following injury, mice received a subcutaneous injection of analgesic buprenorphine (0.25 mg/kg) and maintained at 37 °C until recovery from anesthesia. Mice in the sham group received a craniotomy without controlled cortical impact. VCE-004.8 was dissolved in ethanol:cremophor:saline (1:1:18) and given intraperitoneally at 20 mg/kg starting 1 h after the injury followed by every day until experimental endpoint. The experimental design is shown in Fig. 5A.

#### Rotarod test

The fine motor coordination and learning were assessed using an accelerating rotarod device (Ugo Basile, Gemonio (VA), Italy). Mice were exposed to a period of acclimation and training (first acclimation session: 0 rpm for 30 s followed by three training sessions every 2 days of three trials each: mice were trained at a slow rotational speed (4 rpm/min) for 1 min followed by an accelerating rotational speed (from 4 to 40 rpm in 5 min) with periods of 15 min between trials. One day before injury mice were placed into the apparatus to obtain baseline latency. On each testing day, the mice were exposed to three 300-s accelerating rotarod trials with an inter-trial interval of 30 min. The average latency to the first fall off the rod was recorded. Passive rotation, accompanying the rotating rod without walking, was also considered as a fall.

#### Magnetic resonance imaging (MRI)

For MRI analysis mice (*n* = 5 animals per group) were killed and transcardially perfused with cold paraformaldehyde 4% (PFA 4%). Prior to MRI mouse brains were drained and immersed in a proton-free susceptibility-matching fluid, Fluorinert® FC‐40 (Sigma). Magnetic resonance imaging (MRI) studies were performed at BioImaC (ICTS BioImagen Complutense, Madrid, Spain), node of the ICTS ReDIB (https://www.redib.net/) using 1-T benchtop MRI scanner ICON-1T: Bruker BioSpin GmbH (Ettlingen, Germany). A preliminary study was carried out to optimize MRI protocol in order to select those experiments that would allow damage assessment of the traumatic brain injury (TBI) model. Selected MRI protocol includes the following sequences: fast spin echo (FSE), T1- and T2-weighed, and gradient echo (GE). The gradient echo sequence is very sensitive to magnetic susceptibility caused by the deposition of paramagnetic substances such as blood, allowing it to detect intracranial hemorrhages with high sensitivity. Three-dimensional (3D) images of mouse brains were acquired in axial anatomical orientation with a field of view (FOV) = 16 × 12 × 8 mm and a matrix size 160 × 60 × 40 (resolution 0.1 mm × 0.2 mm × 0.2 mm) for T1&T2 FSE3D and a matrix size 80 × 60 × 40 (resolution 0.2 mm × 0.2 mm × 0.2 mm) for GE. For T1WI3D-FSE images a repetition time (TR) = 1750 ms, an echo time (TE) = 18.75 ms and a number of average (NA) = 3 were used. T2WI3D-FSE parameters were TR = 1750s, echo train length = 8, inter-echo interval = 27 ms (effective TE = 81 ms) and NA = 3. Finally, T1WI3D-GE were acquired with TR/TE = 45/7.5 ms, pulse flip angle = 45° and NA = 8. Total experiment acquisition time ~ 75 min. Axial FSE3D experiments were reconstructed in isotropic coronal orientation (0.1 mm^3^). Although all sequences were used to evaluate the TBI damage, both quantification of edema on T2WI3D-FSE images and 3D reconstructions were using open-source software 3Dslicer v.3.4.0 software [23].

#### Angiogenesis in vivo mouse model

C57BL/6 8 to 10 week-old male mice (*n* = 3 animals per group) were subcutaneously injected with liquid containing Matrigel Gel (Thermo Fischer Scientific) mixed with heparin (0.1 mg/mL) (Ajinomoto Pharma, Tokyo, Japan) [24]. For the positive control recombinant human VEGF-A 165 (200 ng/mL) (R&D Systems) and fibroblast growth factor 2 (1 μg/mL) (R&D Systems) were added to the gel mix. The Matrigel (500 μL) was injected subcutaneously into flanks of mice. VCE-004.8 (20 mg/kg) was i.p. daily administered until experimental endpoint.

#### Tissue processing

For angiogenesis Matrigel plugs were explanted on day 7 and the connective and adipose tissues surrounding the plug were removed and then fixed in 10% formaldehyde and paraffin embedded for histological analysis. For CCI injury model, mice were anesthetized by i.p. administration of ketamine–xylazine and they were transcardially perfused with saline 0.9%. Brains (pericontusional area) were immediately frozen and kept at − 80 °C for quantitative reverse transcriptase-PCR analysis. Other brains were fixed in 4% PFA, washed in 0.1 mL PBS, cryoprotected with a 15% and then a 30% solution of sucrose in 0.1 M PBS, and frozen at -80 ºC. Free-floating brain sections (30 µm thick; Leica Microsystems CM1900 cryostat, Barcelona, Spain) were then processed for immunohistochemistry or immunofluorescence.

#### Immunohistochemistry and immunofluorescence analysis

BBB leaking was assessed by visualization of IgG in the brain. Free-floating sections (30 μm thick) were washed in PBS. Next, endogenous peroxidase activity and nonspecific staining were blocked (5% bovine serum albumin (BSA) plus 5% horse serum). Sections were then incubated in biotinylated anti-mouse IgG antibody (1:500, #BA-2000; Vector Laboratories) overnight at 4 °C. After washing with PBS, sections were incubated with avidin–biotin complex for 30 min (Vectastain® ABC-HRP kit, #PK-4000; Vector Laboratories) and developed using 3,3′-diaminobenzidine (DAB) chromogen (Dako, Santa Clara, CA, USA). All sections were incubated with the same batch of DAB, and all reactions were performed at the same time and exposed for the same amount of time. For leukocyte and macrophage cells infiltration analysis, free-floating brain sections were washed with 0.1 M phosphate buffer (PB). Endogenous peroxidase activity was inhibited with 3.3% hydrogen peroxide in methanol. The sections were blocked with 2.5% normal horse serum and then incubated overnight at 4 ºC in blocking buffer with an anti-CD3 antibody (1:50 Santa Cruz Biotechnology, Santa Cruz, CA, USA), anti-myeloperoxidase (MPO) antibody (1:100, #ab208670, Abcam), anti-F4/80 antibody (1:50, #MCA497, Bio-Rad Laboratories). To block nonspecific binding by endogenous mouse IgG rodent block M (#RBM961, Biocare Medical, Concord, CA, USA) was used prior to anti-CD3 antibody. After incubation with the appropriate secondary antibody, slides were incubated with the avidin–biotin complex for 30 min and then developed with DAB chromogen. All immunohistochemistry images were acquired using a Leica DM2000 LED microscope and were photographed, digitalized using a Leica DFC420c camera. In the case of immunofluorescence analysis, 30-μm-thick free-floating brain sections or 5-μm-thick sections of formalin fixed paraffin embedded Matrigel plugs samples were boiled for 10 min in sodium citrate buffer (10 mM, pH 6.0) for antigen retrieval. The sections were washed three times in PBS containing 0.1% Triton X-100 (Sigma). Nonspecific antibody-binding sites were blocked for 1 h at room temperature with 3% BSA in PBS (Sigma). Next, the sections were incubated overnight at 4 ºC with the following primary antibodies diluted in PBS with 3% BSA. To determine angiogenesis in vivo Matrigel plug samples were incubated with anti-α-Smooth Muscle Actin (α-SMA) (1:500, #53–9760-80; Invitrogen) and anti-CD31 (1:100, #ab28364, Abcam) antibodies. The next day sections were washed three times during 10 min with a wash buffer and incubated in darkness at room temperature for 1 h using anti-rabbit Texas Red (1:100; #A-6399; Thermo Fischer Scientific). To determine glial reactivation, microglia cells were stained with an anti-ionized calcium binding adaptor molecule 1 (Iba-1) antibody (1:100; #019–19,741, Wako Chemical Pure Industry, Osaka, Japan), astrocytes were stained with an anti-glial fibrillary acidic protein (GFAP) antibody (1:100, #sc-33673, Santa Cruz Biotechnology). Neuronal cells were stained with an anti-NeuN antibody (1:100, #MAB377, Millipore). To study BBB integrity, insert sections from TEER experiments or brain sections were incubated overnight at 4 °C with the following primary antibodies: a rabbit anti-VCAM1 (1:100, #ab134047, Abcam) a rabbit or mouse-anti-CD31 (1:100, #ab28364, Abcam or 1:100, #14–0311-85, Invitrogen), rabbit anti-Ki67 (1:100, # ab15580, Abcam), rabbit anti-Zonula Occludens-1 (ZO-1 (1:100, #10,222,233, Invitrogen), mouse anti-claudin-5 (CLDN5) (1:50, #35–2500, Invitrogen). To study PP2A-B55α expression a rabbit anti-PP2A-B55α was used (1:100; Cat: #4953, Cell Signaling Technology). In all studies, after washing, sections were incubated with the appropriate secondary antibody for 1 h at room temperature in the dark. The slides were then mounted using Vectashield Antifade Mounting Medium with DAPI (Vector Laboratories). All immunofluorescence images were acquired using a spectral confocal laser-scanning microscope LSM710, (Zeiss) with a 20×/0.8, 25, 40, or 63×/0.8 Plan-Apochromat oil immersion lens (Advanced Microscopy facility at Maimonides Biomedical Research Institute of Córdoba). All images were quantified in randomly chosen fields using ImageJ software (http://rsbweb.nih.gov/ij/).

#### Detection of apoptosis

Terminal deoxynucleotidyl transferase mediated dUTP nick 3′-end labeling (TUNEL) assay was performed according to the manufacturer’s protocol (Cat. No. 11 684 795 910; Sigma). Tissue sections were incubated with the TUNEL reaction mixture in a humidifier chamber for 1 h at 37 °C in the dark. The slides were then mounted using Vectashield Antifade Mounting Medium with DAPI (Vector Laboratories). All images were acquired using a spectral confocal laser-scanning microscope LSM710, (Zeiss) with a 20×/0.8, 25, 40, or 63×/0.8 Plan-Apochromat oil immersion lens and quantified in randomly chosen fields using ImageJ software (http://rsbweb.nih.gov/ij/).

#### Statistical analysis

All the in vitro data are expressed as the mean ± SD and all the in vivo data are expressed as the mean ± SEM. One-way analysis of variance (ANOVA) followed by the Tukey’s post hoc test for parametric analysis or Kruskal–Wallis post hoc test in the case of non-parametric analysis tests were used to determine the statistical significance. The level of significance was set at *p *˂ 0.05. Statistical analyses were performed using GraphPad Prism version 9 (GraphPad, San Diego, CA, USA).

## Results

### VCE-004.8 inhibits prolyl hydroxylase domain protein 2 (PHD2) Ser125 phosphorylation through a PP2A/B55α pathway

**Fig. 1:**
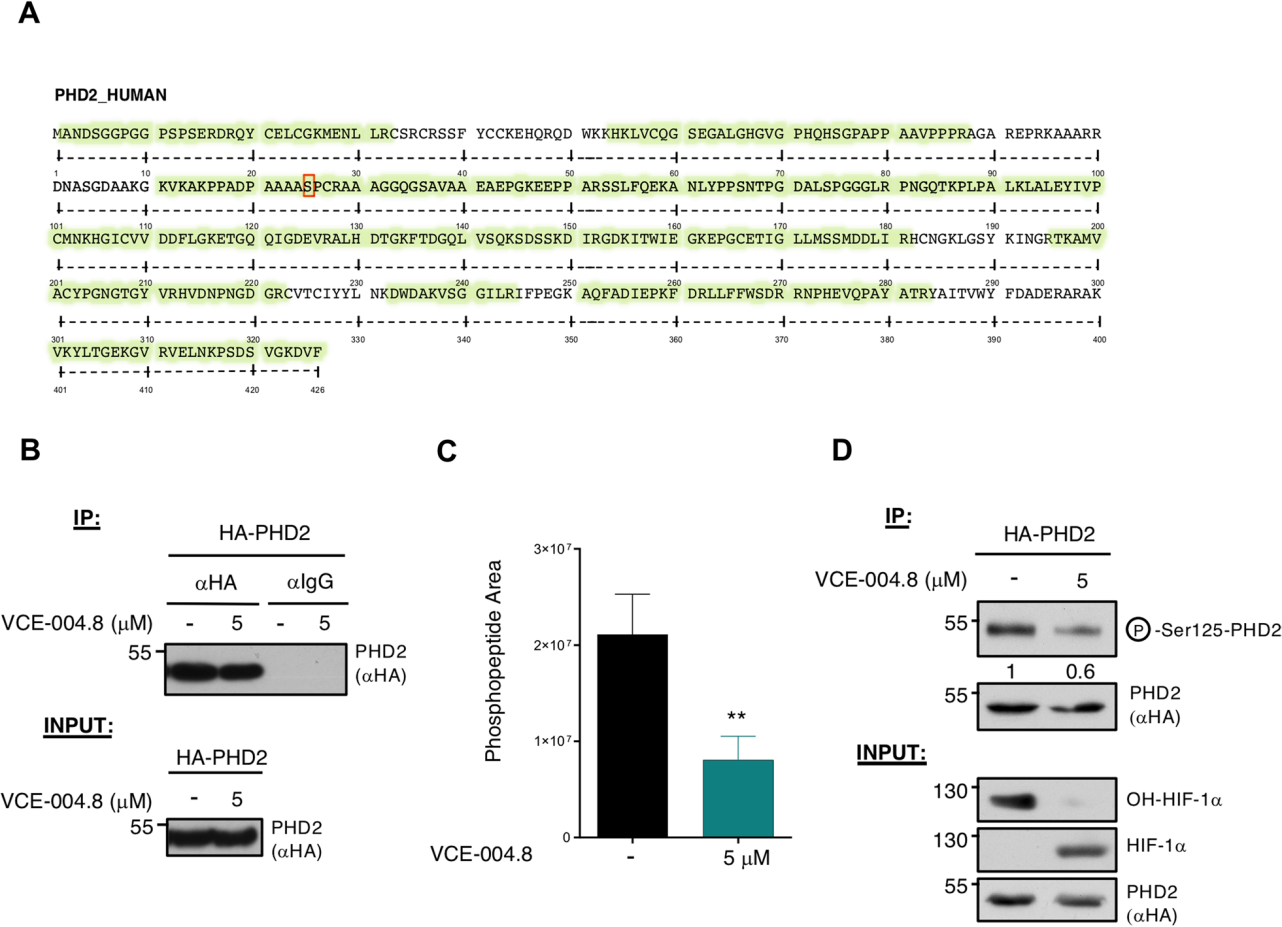
VCE-004.8 modulates PHD2 Ser125 phosphorylation. **A** Schematic representation of mass spectrometry (MS) analysis results. A red box within the PHD2 sequence (101–150) points out phosphorylation found at Ser125 in control experiment, which reduces its signal under VCE-004.8 treatment. **B** HEK-293T cells were transfected with HA-PHD2 plasmid, treated with 5 μM VCE-004.8 for 24 h and finally lysed. PHD2 protein was immunoprecipitated using either specific antibody (anti-HA) or an unspecific one (rat IgG). Immunoprecipitation representation of the proteomic analyses performed (*n* = 3). **C** Immunoprecipitation representation of the proteomic analyses, Ser125 phosphopeptide peak area of the three replicates of the mass spectrometry assay. Data represent the mean ± SD (*n* = 3) and significance was determined by Unpaired *t* test. *p* = 0.0096; **p < 0.01 VCE-004.8 vs Negative control. **D** HEK-293T cells were transfected with HA-PHD2 plasmid, treated with 5 µM VCE-004.8 for 24 h and lysed. A fraction was subjected to IP using an anti-HA antibody. After elution phosphorylation was revealed with a specific anti-phospho Ser125-PHD2 antibody, while exogenous HA-PHD2 protein levels were visualized with an anti-HA antibody (top panel). The remaining extract fraction was tested to analyze the steady state levels of HIF-1α and its hydroxylated form (lower panel). A representative western blot of three independent analyses is shown

**Fig. 2:**
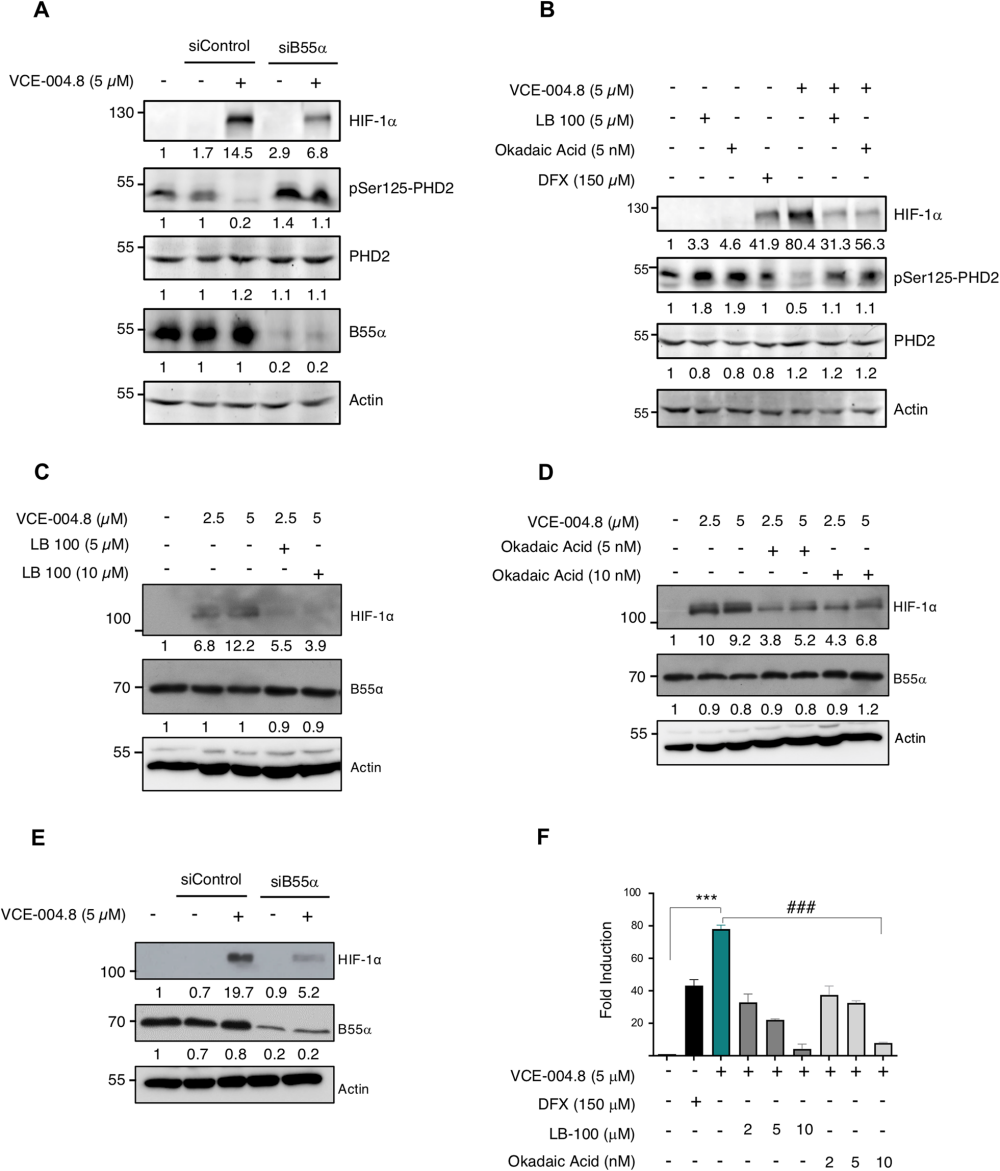
VCE-004.8 activates HIF-1α through a PP2A/B55α pathway. **A** HEK-293T cells were transfected with B55α or scrambled (siControl) siRNAs, after 2 days in culture treated with VCE-004.8 5 μM for 6 h, and protein expression was analyzed by immunoblotting. **B** HEK-293T cells were preincubated with the indicated concentrations of either LB-100 or OA for 30 min and then stimulated for 6 h with VCE-004.8. The protein expression was detected by western blot. **C**, **D** HMEC-1 cells were preincubated with the indicated concentrations of either LB-100 (**C**) or OA (**D**) and stimulated for 3 h with VCE-004.8. The expression of HIF-1α and B55α was detected by western blot. **E** HMEC-1 cells were transfected with B55α or scrambled (siControl) siRNAs, after 2 days in culture treated with VCE-004.8 5 μM for 3 h, and B55α and HIF-1α protein expression was analyzed by immunoblotting. In all the cases a representative western blot of three independent experiments is shown **F** NIH3T3-EPO-luc were stimulated with the indicated concentrations of VCE-004.8, LB-100 and OA for 7 h, DFX was used as a positive control for HRE/EPO-luc transactivation, and luciferase activity measured in the cell lysates. Data represent the mean ± SD (*n* = 3) and significance was determined by Unpaired *t* test. ***p < 0.0001 VCE-004.8 vs Negative control; ###*p* < 0.001 VCE-004.8 + LB-100 vs VCE-004.8 treated cells; ###*p* < 0.0001 VCE-004.8 + OA vs VCE-004.8 treated cells

We have previously described that VCE-004.8 showed PHD2 inhibitory activity [19]. Now, to further investigate the mechanism of action of VCE-004.8 on this pathway, we firstly analyzed the effect on the enzymatic activity using recombinant PHDs. We found that dimethyloxalylglycine (DMOG) but not VCE-004.8 inhibited the enzymatic activity of PHD2, as well as other PHDs (Additional file 1: Figure S1). Secondly, we explored the post-translational modifications (PTMs) of PHD2 after the treatment with the compound. To do that we immunoprecipitated PHD2 from cells after the treatment with VCE-004.8 (Fig. 1B), and PTMs were analyzed by LC–MS/MS and we could identify peptides covering up to a 78% of PHD2 sequence (Fig. 1A). Approximately 60% inhibition in the levels of Ser125 phosphorylation was detected after treatment with VCE-004.8 compared to untreated cells (Fig. 1C). Next, we evaluated the phosphorylation status using a phospho-specific antibody for Ser125. As depicted in Fig. 1D, the treatment with VCE-004.8 clearly reduced the Ser125 phosphorylation levels (around 50% inhibition). It has been described that PP2A/B55α is a relevant modulator of Ser125 PHD2 [12]. To determine whether VCE-004.8 modifies Ser125 PHD2 phosphorylation through PP2A/B55α, we carried out a loss-of-function experiment. As expected, B55α depletion by siRNA in HEK-293T prevented Ser125 PHD2 dephosphorylation and reduced HIF-1 stabilization in cells treated with VCE-004.8 (Fig. 2A). Moreover, siB55α also reduced HIF-1α stabilization in VCE-004.8-treated HMEC-1 cells (Fig. 2E). In parallel, we used two well-known pan-PP2A inhibitors, LB-100 and okadaic acid (OA), which inhibited VCE-004.8-induced Ser125 PHD2 dephosphorylation and HIF-1α expression (Fig. 2B). We also show that LB100 and OA impaired HIF-1α stabilization in HMEC-1 treated with VCE-004.8 (Fig. 2C, D). Furthermore, this observation was corroborated determining the downstream effects of HIF-1α (HRE-EPO gene promoter induction) in the presence of both inhibitors. As shown in Fig. 2F, HIF-1α transcriptional activity induced by VCE-004.8 was inhibited up to 90% by increasing LB-100 or OA in a concentration-dependent manner.

### VCE-004.8 induces angiogenesis in vivo and prevents in vitro BBB disruption

**Fig. 3:**
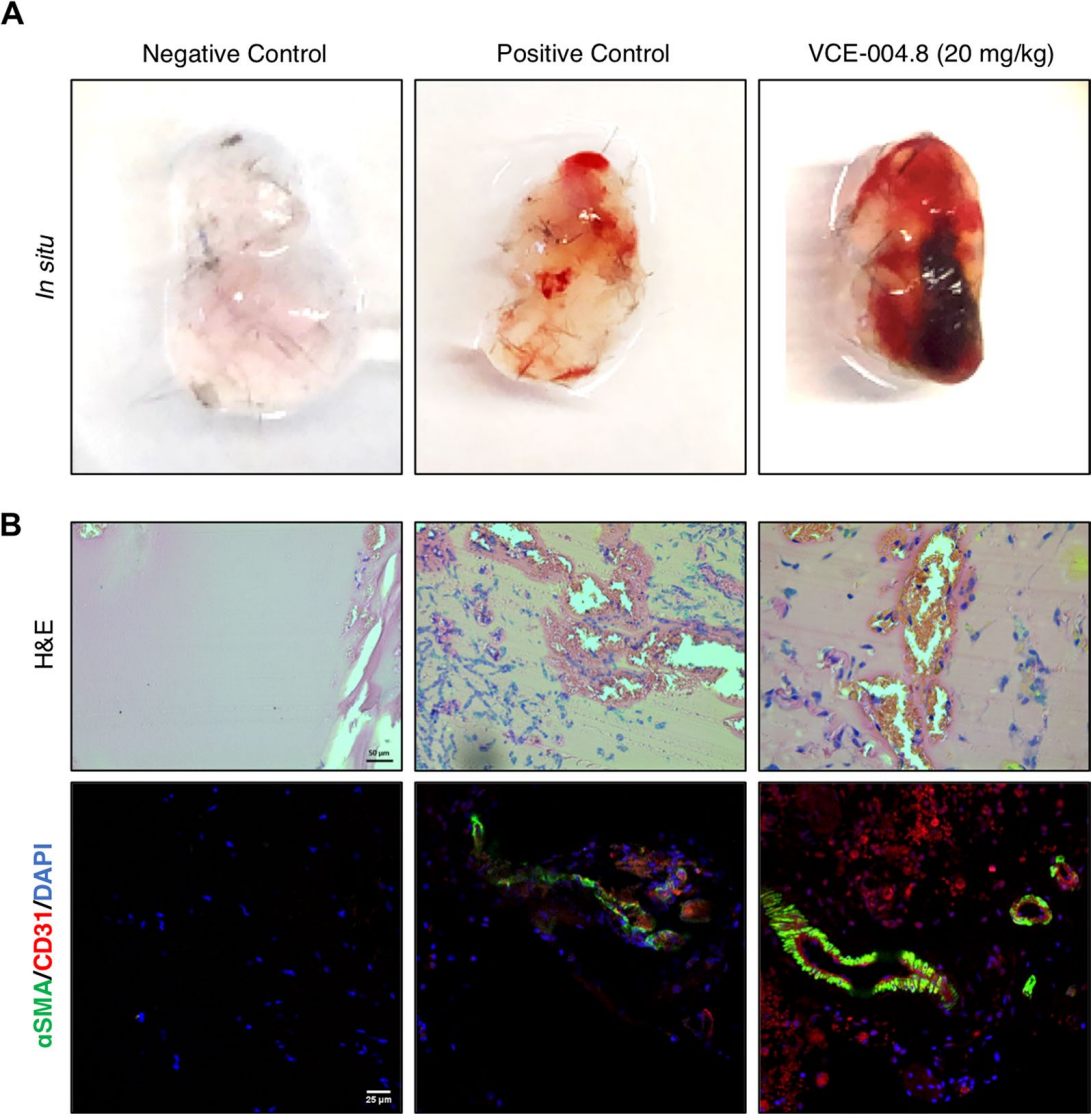
VCE-004.8 induces angiogenesis in vivo. **A** Gross morphology of Matrigel in situ. **B** Induction of vascularization shown by H&E and immunostaining of CD31^+^/αSMA^+^ cells in the plugged Matrigel. Negative control corresponds to Matrigel that only contains heparin; positive control corresponds to Matrigel that contains heparin plus VEGFA and FGF2. Scale bars equivalent to 50 µm (H&E) and 25 μm (CD31^+^/αSMA^+^)

Since we have previously described that VCE-004.8 induces angiogenesis in vitro as a functional consequence on HIF-1α stabilization [19], we wanted to explore this activity in vivo. To examine the angiogenic potential of VCE-004.8, a Matrigel plug was implanted subcutaneously into mice. As shown in Fig. 3A, Matrigel without treatment (negative control) is light pink. However, the plug treated with VCE-004.8 is dark red. This observation could imply a functional vasculature and greater density blood vessel in the Matrigel plugs treated with VCE-004.8. To confirm this, we identified endothelial cells through the double staining α-SMA and CD31. The results demonstrated that VCE-004.8 at 20 mg/kg significantly increased the formation of functional vessel (CD31/α-SMA) after 7 days of Matrigel implantation (Fig. 3B).

**Fig. 4:**
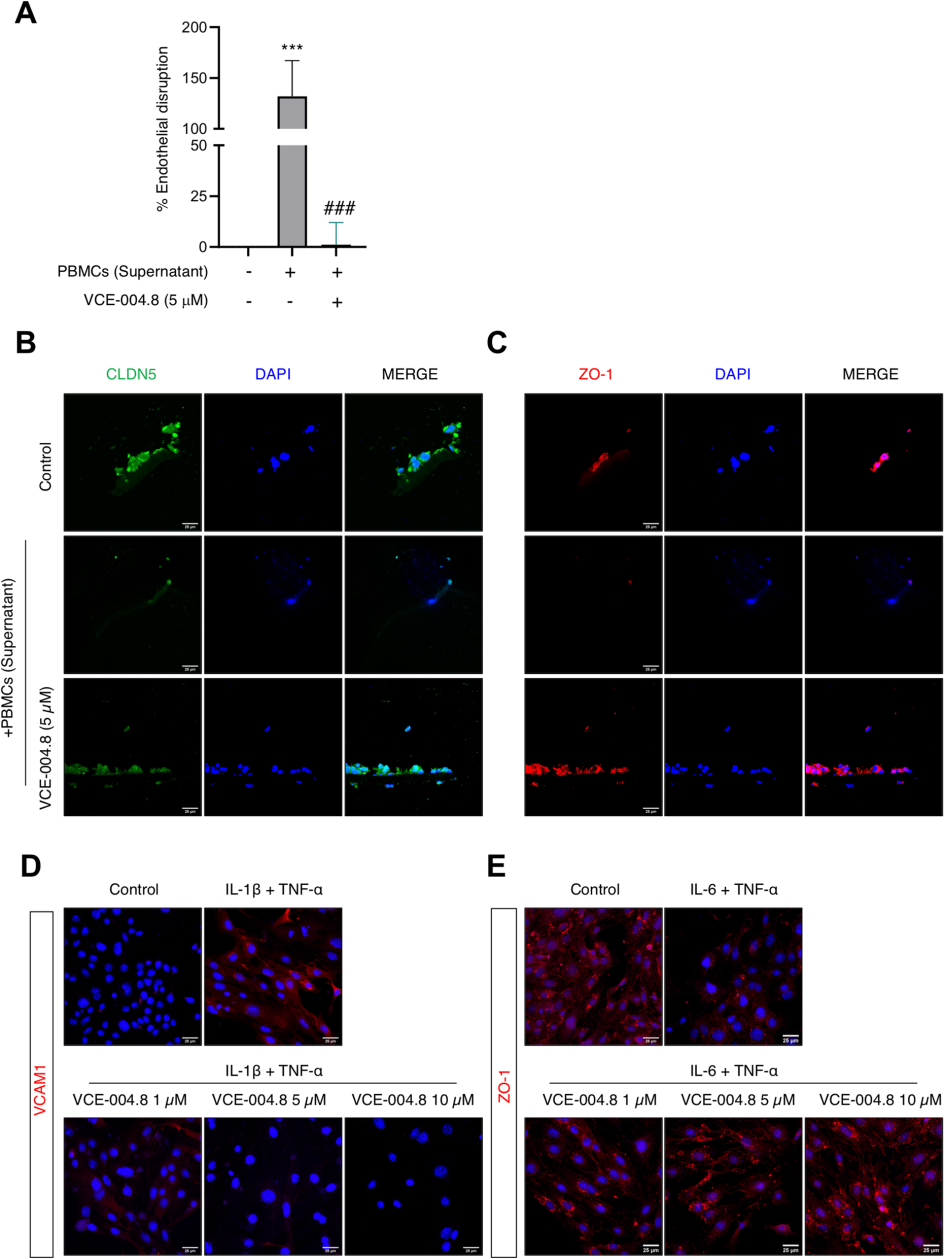
VCE-004.8 prevents BBB disruption in vitro. **A** VCE-004.8 treatment protected against endothelial disruption promoted by PBMCs supernatant in the bEnd5 cell line. Data represent the mean ± SD (*n* = 3) and significance determined by one-way ANOVA followed by Tukey’s test. *p* = 0.0007; ***p < 0.001 for PBMCs supernatant-treated cells vs control; *p* = 0.0007; ###*p* < 0.001 for VCE-004.8-treated cells vs PBMCs supernatant-treated cells. **B**, **C** Representative fluorescence images of bEnd5 cells cultured in Transwell filters from the TEER assay stained for CLDN5 and ZO-1 tight junction proteins. A representative image of three independent experiments is shown (*n* = 3). Scale bars equivalent to 25 μm. Representative images of immunostaining of VCAM1 (**D**) and ZO-1 (**E**) expression in cultured bEnd5 cells stimulated during 24 h with proinflammatory cytokines. Cells were counterstained with DAPI to identify nuclei. A representative image of three independent experiments is shown. Scale bars equivalent to 25 μm

Next, we studied the effect of this compound on in vitro BBB disruption and endothelial cells inflammation. Firstly, using an in vitro model of BBB where its disruption was induced by a cocktail of proinflammatory cytokines (supernatant from PHA-stimulated human peripheral mononuclear cells; PBMCs supernatant) we measured the transendothelial electrical resistance (TEER) in the bEnd5 cell line. As shown in Fig. 4A, the treatment with VCE-004.8 prevented BBB disruption induced by PBMCs supernatant. In addition, we studied the expression of the two main tight junction proteins in these brain endothelial cells cultured on inserts for the TEER assay at day 13. Immunofluorescence studies revealed the preventive effect of VCE-004.8 on the reduced expression of claudin-5 and ZO-1 caused by PBMCs supernatant (Fig. 4B, C). To confirm these results, we studied ZO-1 and VCAM1 expression in bEnd5 cells under proinflammatory conditions. VCAM1 expression was increased under proinflammatory conditions, which was prevented by the treatment with VCE-004.8 (Fig. 4D). In addition, VCE-004.8 clearly restored the ameliorated expression of ZO-1 in bEnd5 cells treated with TNF-α plus IL-6 (Fig. 4E).

### VCE-004.8 treatment ameliorates early motor deficits after TBI

**Fig. 5:**
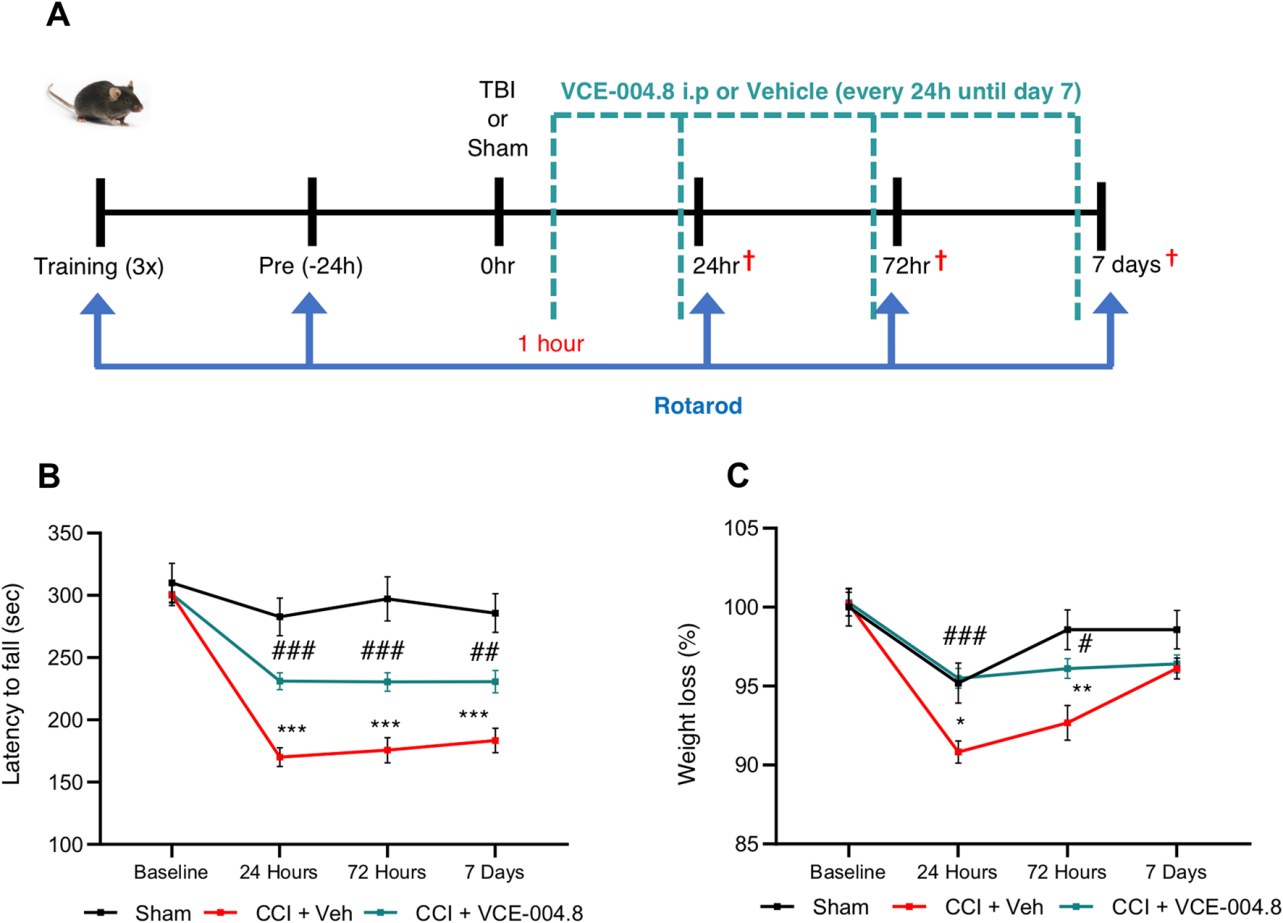
VCE-004.8 treatment protects from TBI-induced motor impairment. **A** Experimental design for CCI. **B** VCE-004.8 administration (20 mg/kg) attenuated the CCI-induced impaired motor performance. Motor recovery was analyzed by Rotarod test prior to and at 24 h, 72 h and 7 days post-CCI. Data are represented as mean ± SEM, and significance was determined by 2-way ANOVA followed by the Tukey’s post hoc test. 24 h, 72 h and 7 days: ***p˂0.0001 CCI + Veh vs Sham; 24 h and 72 h: ###*p* ˂ 0.0001 CCI + VCE-004.8 vs CCI + Veh; 7 Days: *p* = 0.0098; ##*p*˂0.01 CCI + VCE-004.8 vs CCI + Veh. **C** Weight determination in the different groups of mice. Data are represented as mean ± SEM, and significance was determined by 2-way ANOVA followed by the Tukey’s post hoc test. 24 h: *p* = 0.0172; *p < 0.05, 72 h: *p* = 0.0013; **p < 0.01 CCI + Vehicle vs Sham; 24 h ###*p* < 0.001; 72 h *p* = 0.0120; #*p* < 0.05 CCI + VCE-004.8 vs CCI + Vehicle

Since VCE-004.8 is a multifunctional compound playing a key role on a series of in vitro and in vivo crucial targets in neuroinflammation and BBB disruption, we evaluated its efficacy on a traumatic brain injury model where both immediate and delayed dysfunction of the BBB/gliovascular unit and neuroinflammations are the hallmarks of the disease. The therapeutic potential of VCE-004.8 was evaluated in a moderate controlled cortical impact (CCI) injury model where the compound was given intraperitoneally starting 1 h after the injury followed by every day until experimental endpoint (Fig. 5A). To determine the effect of VCE-004.8 treatment on motor dysfunction associated with traumatic brain injury we performed the rotarod test. Mice in the Sham group showed the best performance compared with the other CCI groups. Remarkably, mice that received the treatment with VCE-004.8 ameliorated the loss of motor coordination (up to 60% of recovery). This effect was observed starting at 24 h after the traumatism and was maintained through the study (Fig. 5B). Furthermore, weight loss was monitored in mice as an additional measure of clinical outcome and as shown in Fig. 5C the treatment with VCE-004.8 significantly reduced weight loss at 24 h after the injury maintaining the same weight as the Sham group.

### VCE-004.8 attenuates cerebral edema after TBI

**Fig. 6:**
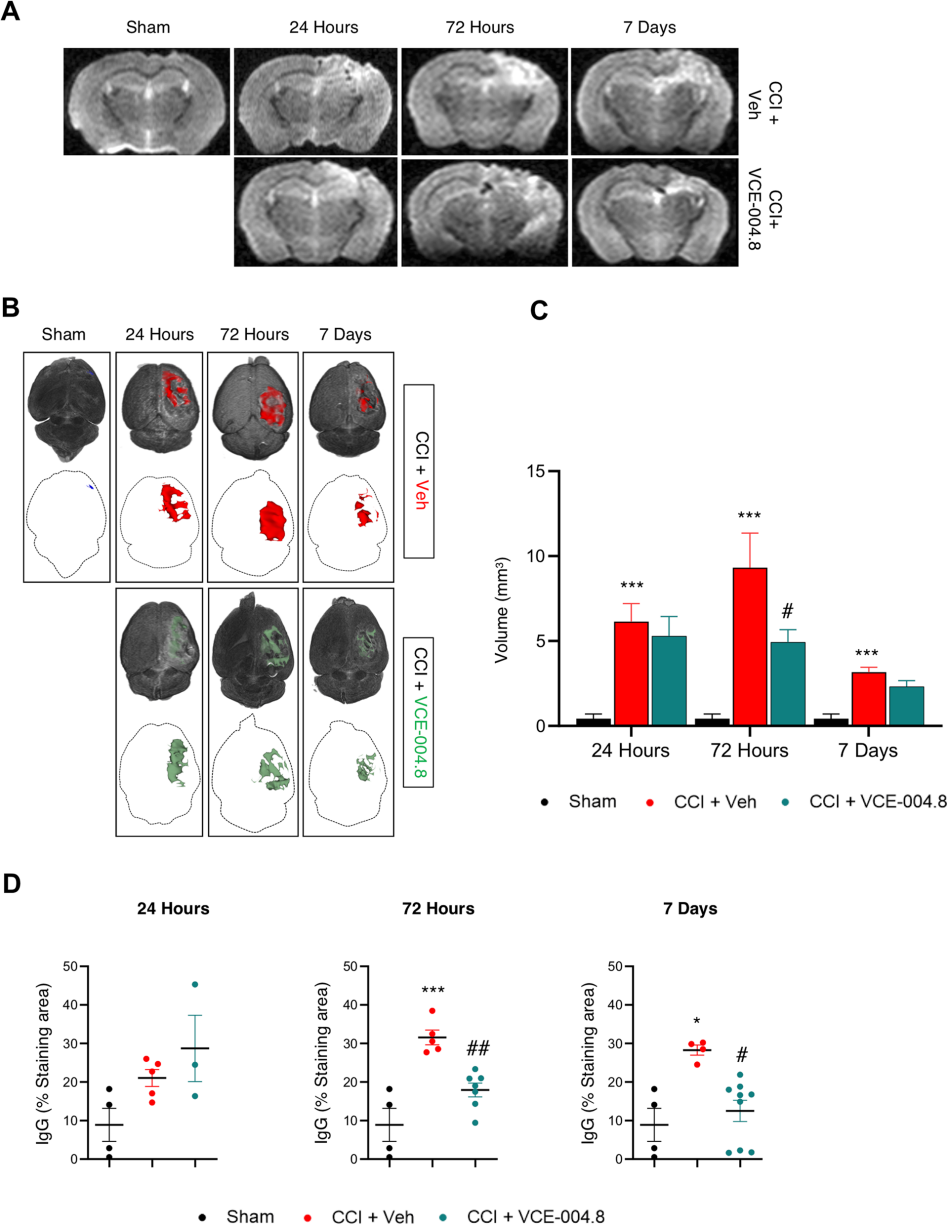
VCE-004.8 attenuates brain edema after TBI. **A** Representative T2-weighted MRI images at 1 to 7 days post-TBI. The damaged area is defined by a hyperintense region over the cortex, indicating edema formation. **B** Top images, 3D rendering of the MRI scans from A is used to show the location and calculate the volume of tissue edema (colored region). Bottom images show edematous tissue only in axial view. **C** Volume quantification of edematous tissue. Data are represented as mean ± SEM, and significance was determined by one-way ANOVA followed by the Tukey’s test. 24 h and 72 h: *p* = 0.0003; ***p < 0.001, 7 days: *p* = 0.0005; *** *p* < 0.001 CCI + Vehicle vs Sham; 72 h: *p* = 0.0359; #*p* < 0.05 CCI + VCE-004.8 vs CCI + Vehicle. **D** Quantification of peripheral IgG infiltration into the brain in pericontusional cortex at 24 h, 72 h and 7 days after the injury. Data are represented as mean ± SEM, and significance was determined by one-way ANOVA followed by the Tukey’s post hoc or Kruskal–Wallis post hoc test. 72 h: *p* = 0.0001; ***p < 0.001, 7 days: *p* = 0.0190: *p < 0.05 CCI + Vehicle vs Sham; 72 h: *p* = 0.0036; ##*p* < 0.01, 7 days: *p* = 0.0265; #*p* < 0.05 CCI + VCE-004.8 vs CCI + Vehicle

To determine TBI-associated edema, MRI studies were carried out. This procedure has been applied in the clinical setting to quantify the severity of contusion edema following brain injury. T2-weighted images were segmented, and their 3D reconstructions and edema volumes were quantified. T2-weighted imaging showed no injury in Sham group (Fig. 6A). In contrast to this observation, in VCE-004.8 and vehicle-treated CCI groups injuries involving the cortical and subcortical areas of the brain were observed (Fig. 6A). The volume of edema was higher after 72 h of the traumatism, but it had been partially resolved 7 days after injury (Fig. 6B, C). Mice that received the treatment with VCE-004.8 showed a clear reduction in the formation of edema at all times studied, this reduction being significant at 72 h (4.93 ± 0.73 mm^3^ in VCE-004.8-treated group vs 9.32 ± 2.03 mm^3^ in vehicle-treated group). The basis of edema is the infiltration of fluid into the cerebral parenchyma as a result of BBB dysfunction [25]. To study the extravasation level, we determined the infiltration in the pericontusional site of peripheral immunoglobulin G (IgG) as a marker of BBB disruption. Immunohistochemical examination showed that there was minimal IgG expression in Sham mice (Fig. 6D and Additional file 1: Figure S2). Nevertheless, immunostaining revealed a significant increase in IgG expression after the injury. Remarkably, VCE-004.8-treated mice group showed a reduced brain extravasation of peripheral IgG after 72 h and 7 days in comparison with vehicle-treated mice (17.93 ± 1.78 (72 h); 12.52 ± 2.73 (7 days) % Staining area in VCE-004.8-treated group vs 31.56 ± 1.91 (72 h); 28.28 ± 1.30 (7 days) % Staining area in vehicle-treated group at the times indicated) (Additional file 1: Figure S2 and Fig. 6D).

### VCE-004.8 treatment preserves BBB integrity after TBI

**Fig. 7:**
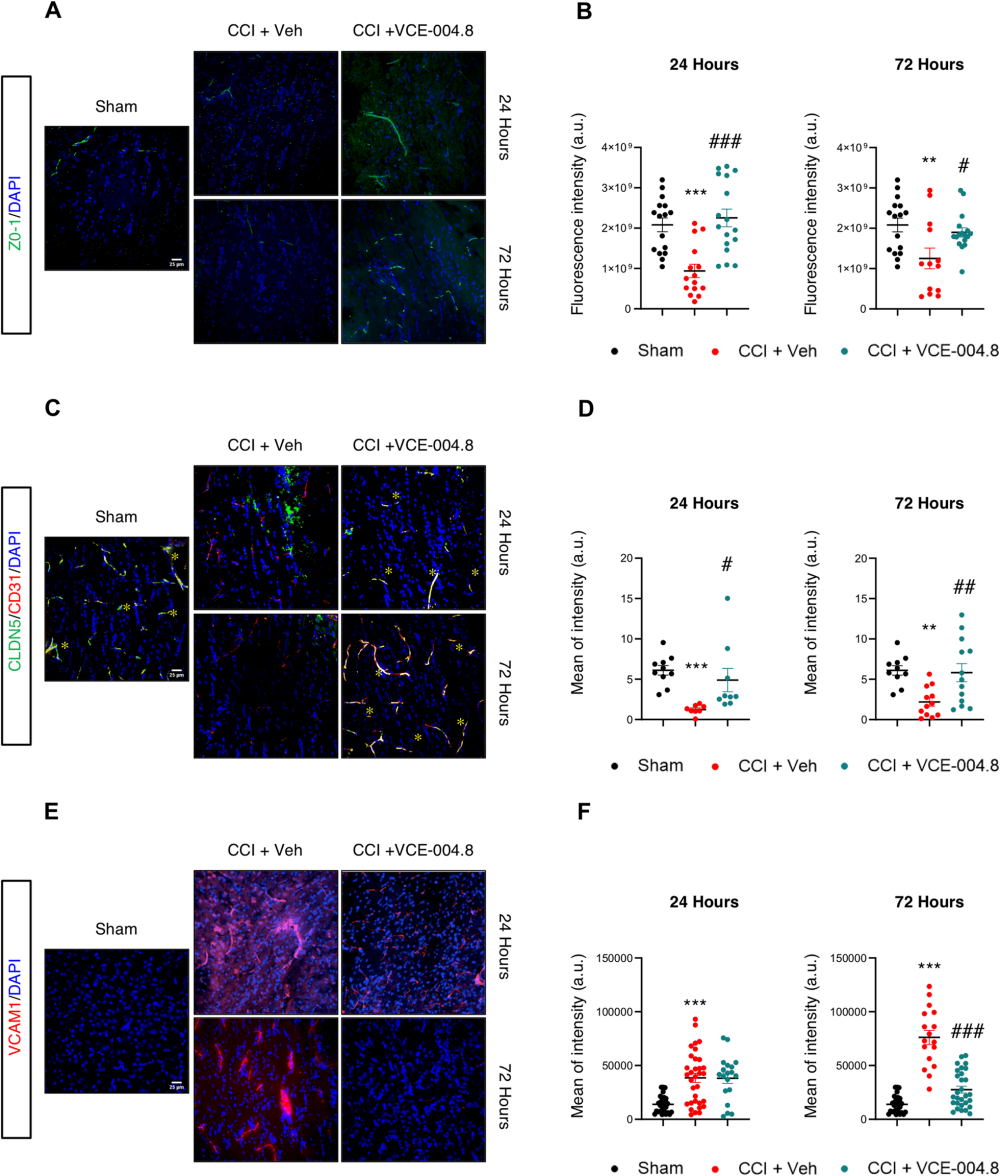
Effect of VCE-004.8 treatment on BBB integrity after TBI. **A**-**C**-**E** Representative immunofluorescence images and quantification **B**-**D**-**F** showing the expression of ZO-1, CLDN5 and VCAM1 in the ipsilateral corpus callosum at 24 h and 72 h after the brain injury. Data are represented as mean ± SEM, and significance was determined by one-way ANOVA followed by the Tukey’s post hoc test or Kruskal–Wallis post hoc test. **B** (24 h: *p* = 0.0003; ***p < 0.001, 72 h: *p* = 0.0073; **p < 0.01 CCI + Vehicle vs Sham; 24 h: *p* < 0.0001; ###*p* < 0.001, 72 h: *p* = 0.0408; #*p* < 0.05 CCI + VCE-004.8 vs CCI + Vehicle), **D** (24 h: *p* < 0.0001; ***p < 0.001, 72 h: *p* = 0.0086; **p < 0.01 CCI + Vehicle vs Sham; 24 h: p = 0.0180; #*p* < 0.05, 72 h: *p* = 0.0090; ##*p* < 0.01 CCI + VCE-004.8 vs CCI + Vehicle), **F** (24 h and 72 h: *p* < 0.0001; ***p < 0.001 CCI + Vehicle vs Sham; 72 h: *p* < 0.0001; ###*p* < 0.001 CCI + VCE-004.8 vs CCI + Vehicle). Scale bars equivalent to 25 µm

Considering the preventive effect of VCE-004.8 on BBB disruption observed in vitro and the positive results obtained from MRI studies, we hypothesized that these effects could be partially due to the maintaining of BBB integrity. To evaluate this hypothesis, we investigated the effect of VCE-004.8 in the expression of BBB tight junction proteins such as ZO-1 and CLDN-5 in the ipsilateral corpus callosum. As depicted in Fig. 7A, B the brain injury induced a loss of ZO-1 expression and the treatment with VCE-004.8 ameliorated this structural loss measured after 24 and 72 h of injury (2.25 × 10^9^ ± 2.19 × 10^8^ (24 h); 1.90 × 10^9^ ± 1.14 × 10^8^ (72 h) fluorescence intensity in VCE-004.8-treated group vs 9.39 × 10^8^ ± 1.65 × 10^8^ (24 h); 1.25 × 10^9^ ± 2.58 × 10^8^ (72 h) fluorescence intensity in vehicle-treated group at the times of study). Similarly, the expression of CLDN-5 was restored in the VCE-004.8-treated mice group versus the vehicle-treated mice group (4.89 ± 1.45 (24 h); 5.83 ± 1.11 (72 h) mean of intensity in VCE-004.8-treated group vs 1.24 ± 0.20 (24 h); 2.18 ± 0.52 (72 h) mean of intensity in vehicle-treated mice at the times indicated) (Fig. 7C, D). Furthermore, we were interested in determining the expression of VCAM1. Figure 7E, F shows that VCAM1 was highly expressed in the ipsilateral corpus callosum of vehicle-treated mice (38,527 ± 4275 (24 h); 76,128 ± 6580 (72 h) mean of intensity in vehicle-treated mice vs 13,881 ± 1510 mean of intensity in Sham group at both times). In contrast, animals that received VCE-004.8 treatment showed a significant reduced expression of VCAM1 in the same area (27,600 ± 3157 mean of intensity in VCE-004.8-treated group vs 76,128 ± 6580 mean of intensity in vehicle-treated mice at 72 h) (Fig. 7E, F).

**Fig. 8:**
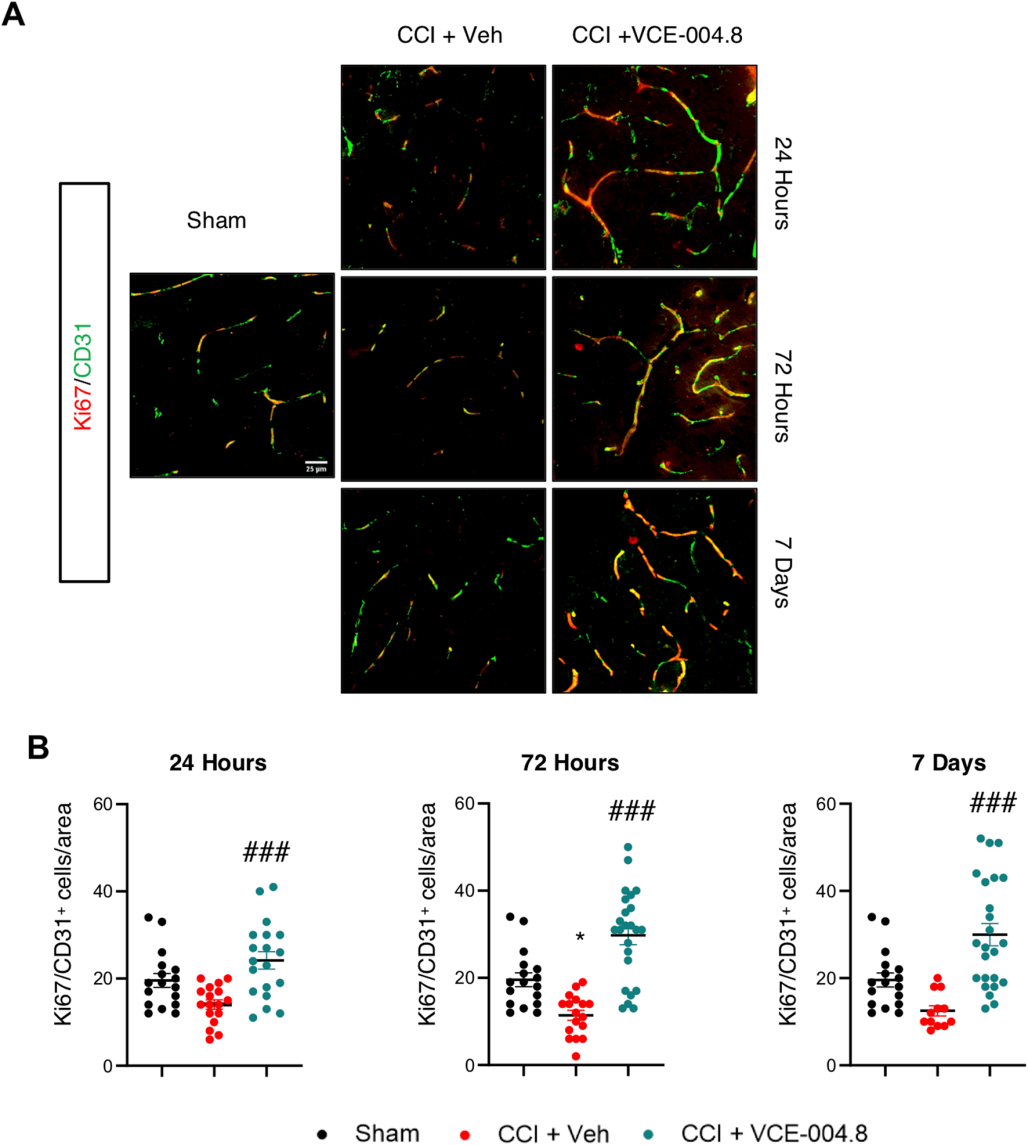
VCE-004.8 upregulates angiogenesis after TBI: endothelial cell proliferation. **A** Representative immunofluorescence images for CD31 and Ki67 expression in the pericontusional cortex at 24 h, 72 h and 7 days after the brain injury. Scale bar equivalent to 25 µm. **B** The quantification of Ki67^+^/CD31^+^ cells per area is represented as mean ± SEM, and significance was determined by one-way ANOVA followed by the Tukey’s post hoc test. 24 h: *p* = 0.0002; ###*p* < 0.001, 72 h and 7 days: *p* < 0.0001; ###*p* < 0.001 CCI + VCE-004.8 vs CCI + Vehicle

Endothelial cells play a significant role in angiogenesis, and to improve their activity creating the collateral circulation after the brain injury is necessary. Thus, we wanted to investigate the role of VCE-004.8 in angiogenesis after the brain injury. To do this the endothelial cell proliferation in the pericontusional area was examined by identifying the expression of CD31/Ki67 markers. Mice treated with vehicle after CCI showed a reduction in the number of CD31^+^/Ki67^+^ endothelial cells after 24 h, 72 h and 7 days of brain injury. Interestingly, the results showed that the presence of CD31^+^/Ki67^+^ endothelial cells was higher in the VCE-004.8-treated mice group than in mice which only received vehicle (24.16 ± 2.00 (24 h); 29.75 ± 2.14 (72 h); 29.96 ± 2.55 (7 days) CD31^+^/Ki67^+^ cells/area in VCE-004.8-treated group vs 14.06 (24 h) ± 1.06; 11.41 ± 1.14 (72 h); 12.50 ± 1.17 (7 days) CD31^+^/Ki67^+^ cells/area in vehicle-treated mice at the times indicated) (Fig. 8A, B). Since endothelial protection and vascular remodeling for the PP2A/B55α complex have been described, we wondered the effect of VCE-004.8 on this phosphatase complex after TBI. As shown in Additional file 1: Figure S5A and S5B, animals treated with VCE-004.8 showed a significant increase in PP2A/B55α phosphatase complex expression that colocalized with CD31 in the pericontusional area at least 72 h after TBI (1,852,639 ± 290,512 mean of Integrated density in VCE-004.8-treated group vs 841,594 ± 191,316 mean of Integrated density in vehicle-treated mice at 72 h). Since an increase of PP2A/B55α expression would imply a higher stabilization of HIF-1α, we studied the expression of HIF-1α-dependent genes such as Vegf and Epo in the pericontusional cortex. VCE-004.8 significantly enhanced the expression of Vegf and showed a trend to induce the expression of Epo (Additional file 1: Figure S6).

### VCE-004.8 treatment attenuates neuroinflammation

**Fig. 9:**
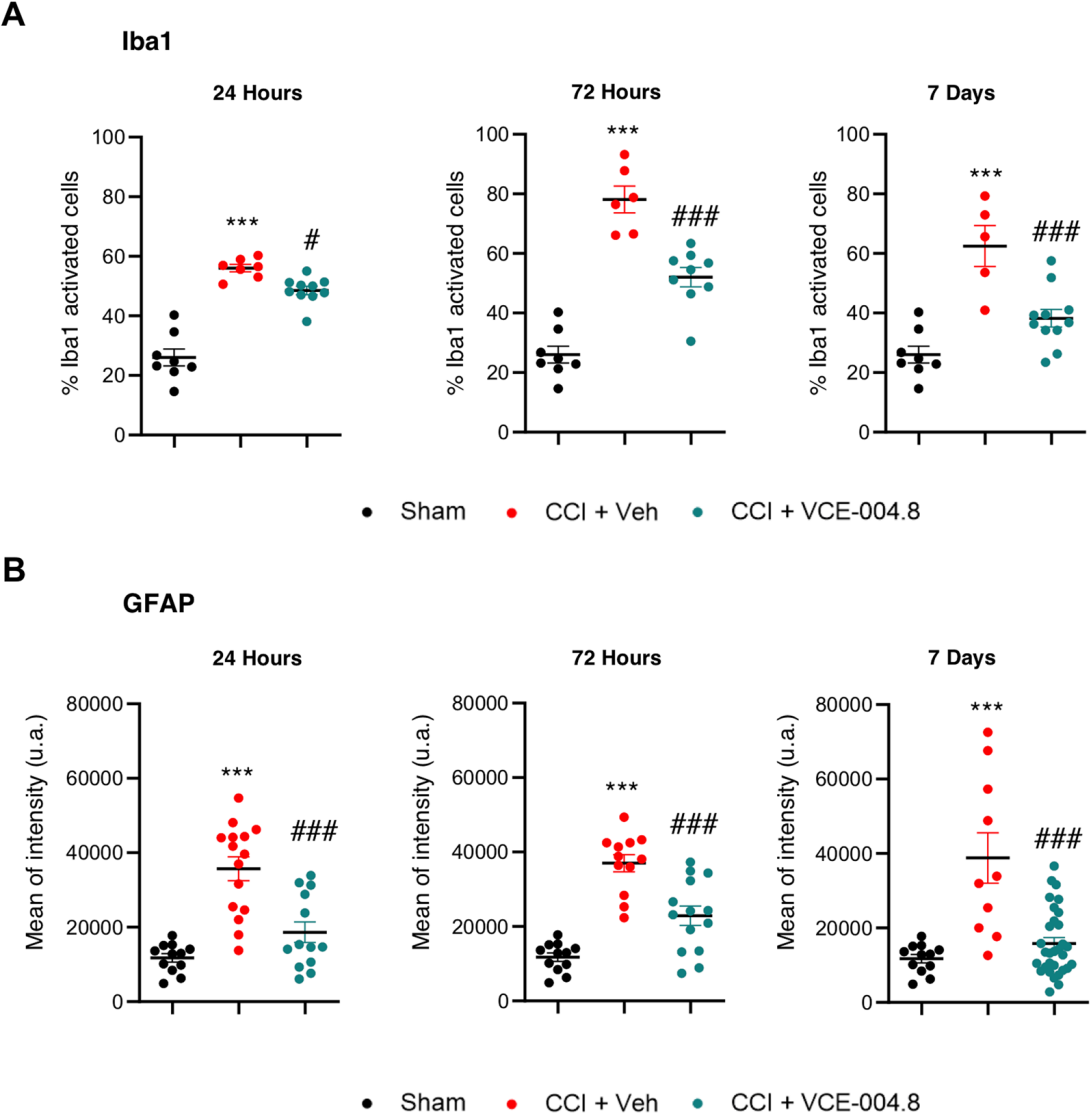
VCE-004.8 ameliorates microglia and astrocyte activation after TBI. **A** Microglial and **B** astrocyte reactivity (Iba1 + cells and GFAP + cells, respectively) in the pericontusional cortex (Iba1) and corpus callosum (GFAP). Graphs represent the quantification of confocal microscopy images for each marker at 24 h, 72 h and 7 days. Data are represented as mean ± SEM, and significance was determined by one-way ANOVA followed by the Tukey’s post hoc test. **A** (24 h, 72 h and 7 days: *p* < 0.0001; ***p < 0.001 CCI + Vehicle vs Sham, 24 h: *p* = 0.0347; #*p* < 0.05, 72 h: *p* = 0.0001; ###*p* < 0.001, 7 days: *p* = 0.0010; ###p < 0.001 CCI + VCE-004.8 vs CCI + Vehicle), **B** (24 h, 72 h and 7 days: *p* < 0.0001; ***p < 0.001 CCI + Vehicle vs Sham, 24 h and 72 h: *p* = 0.0001; ###*p* < 0.001, 7 days: *p˂0*.0001; ###*p* < 0.001 CCI + VCE-004.8 vs CCI + Vehicle)

Neuroinflammation is a major component of the secondary damage after TBI. A reactive state of glia cells, predominantly microglia, represents a marker of chronic immune activation linked to the most neurodegenerative diseases. Thus, we investigated the effect of VCE-004.8 on glial activation by staining Iba-1^+^ and GFAP^+^ cells in pericontusional cortex and corpus callosum, respectively. Histopathological analysis showed low expression levels of activated cells in Sham mice, but vehicle-treated mice showed microglial and astrocytic activation, which was significantly ameliorated by VCE-004.8 treatment (Additional file 1: Figure S3A and 3B). Quantitative assessment by determining the solidity shape descriptor, which provides information on the ameboid shape [26], showed a significant increase in the percentage of activated Iba1^+^ cells (an increase in the percentage of cells with > 0.5 solidity value) after the brain injury. Furthermore, a higher GFAP fluorescence intensity was observed after the injury. As shown in Fig. 9A, the microglia activation was greatly reduced by VCE-004.8 treatment during the study (48.57 ± 1.40 (24 h); 52.04 ± 3.22 (72 h); 38.23 ± 2.96 (7 days) % Iba1 activated cells in VCE-004.8-treated group vs 56.00 ± 1.25 (24 h); 78.15 ± 4.49 (72 h); 62.49 ± 6.87 (7 days) % Iba1 activated cells in vehicle-treated mice at the times studied). In the same manner and as shown in Fig. 9B, astrocytic reactivation was ameliorated by VCE-004.8 treatment (18,646 ± 2768 (24 h); 22,876 ± 2306 (72 h); 15,819 ± 1592 (7 days) mean of intensity in VCE-004.8-treated group vs 35,697 ± 2768 (24 h); 37,026 ± 2306 (72 h); 38,805 ± 6787 (7 days) mean of intensity in vehicle-treated mice at the times indicated).

### Effects of VCE-004.8 on brain immune cell infiltration

**Fig. 10:**
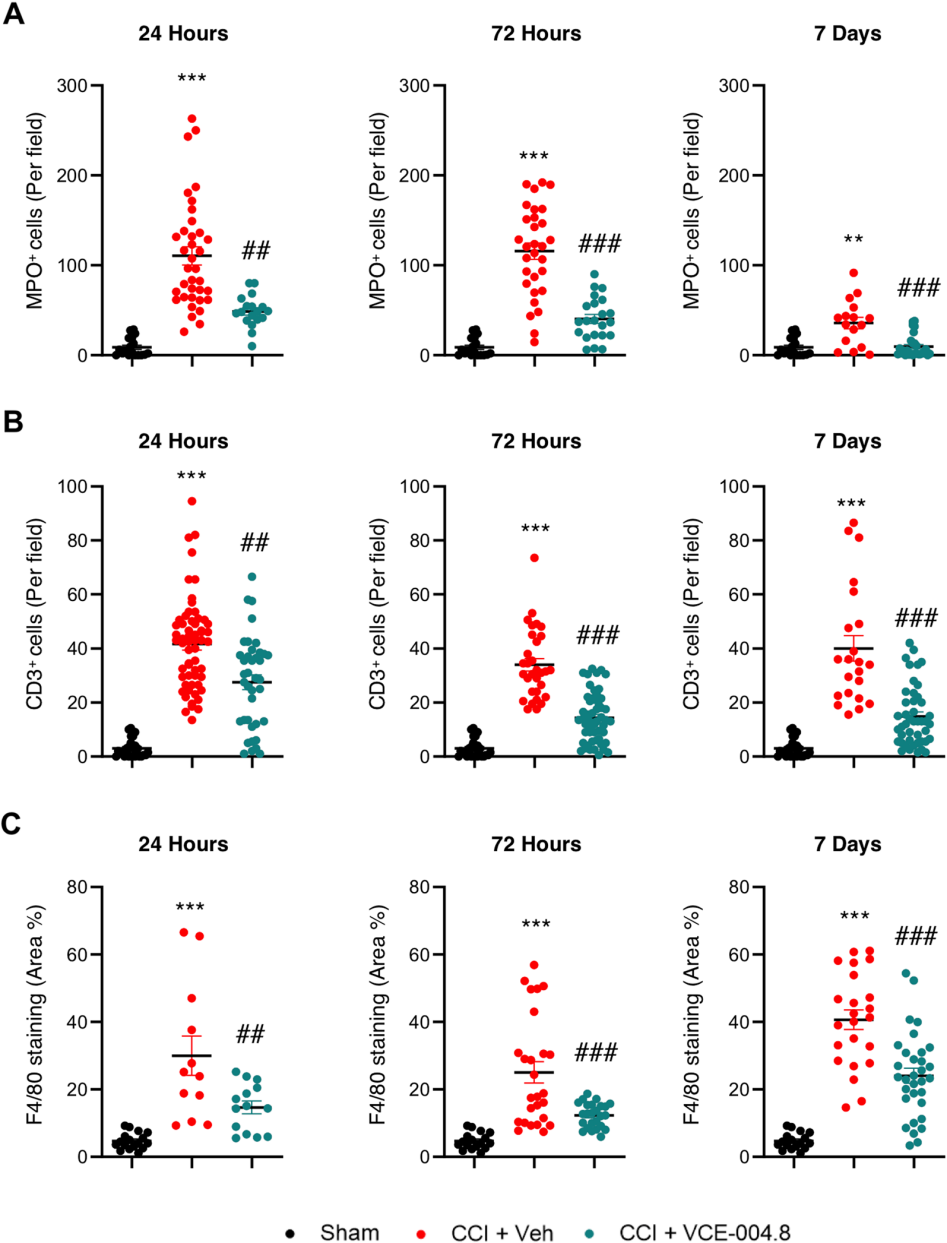
VCE-004.8 reduces leukocyte and macrophage cell infiltration after TBI. (**A**-**B**) VCE-004.8 reduced leukocyte invasion in the pericontusional cortex post-CCI. Graphs represent the cell count analysis of neutrophils (MPO^+^ cells) and T cells (CD3^+^ cells) of microscopy images for each marker at 24 h, 72 h and 7 days. Data are represented as mean ± SEM, and significance was determined by one-way ANOVA followed by the Tukey’s post hoc test or Kruskal–Wallis post hoc test. **A** (24 h and 72 h: *p* < 0.0001; ***p < 0.001, 7 days: *p* = 0.0011; **p < 0.01 CCI + Vehicle vs Sham, 24 h: *p* = 0.0023; ##*p* < 0.01, 72 h: *p* < 0.0001; ###*p* < 0.001, 7 days: *p* = 0.0010; ###*p* < 0.001 CCI + VCE-004.8 vs CCI + Vehicle), **B** (24 h, 72 h and 7 days: *p* < 0.0001; ***p < 0.001 CCI + Vehicle vs Sham, 24 h: *p* = 0.0055; ##*p* < 0.01, 72 h: *p* < 0.0001; ###*p* < 0.001, 7 days: *p* = 0.0002; ###*p* < 0.001 CCI + VCE-004.8 vs CCI + Vehicle). **C** VCE-004.8 ameliorated macrophage cell infiltration in the pericontusional cortex post-CCI. Graphs represent the quantification of immunolabeled images with F4/80 marker. Data are represented as mean ± SEM, and significance was determined by one-way ANOVA followed by the Tukey’s post hoc test or Kruskal–Wallis post hoc test. 24 h, 72 h and 7 days: *p* < 0.0001; ***p < 0.001 CCI + Vehicle vs Sham, 24 h: *p* = 0.0043; ##*p* < 0.01, 72 h and 7 days: *p* < 0.0001; ###*p* < 0.001 CCI + VCE-004.8 vs CCI + Vehicle

BBB disruption associated with TBI allows an entry of inflammatory factors and cells to the brain parenchyma, leading the progression of secondary injuries following the damage. Immunohistochemical studies of brain sections for neutrophils (Myeloperoxidase positive cells, MPO^+^) and T cells (CD3^+^) showed that TBI led to the extravasation of leukocytes that were predominantly located in the pericontusional area (Additional file 1: Figure S4A, S4B). We found that VCE-004.8 treatment significantly reduced the presence of leukocyte cells in the pericontusional region from 24 h after the injury compared with those in the vehicle-treated mice group (Additional file 1: Figure S4A, 4B). Quantification showing the count analysis of neutrophils and T cells is represented in Fig. 10A, B, respectively. In the case of MPO^+^ cells the quantification showed: 48.72 ± 4.13 (24 h); 40.32 ± 5.04 (72 h); 9.57 ± 2.39 (7 days) MPO^+^ cells (per field) in VCE-004.8-treated group vs 110.5 ± 10.03 (24 h); 115 ± 9.12 (72 h); 35.78 ± 6.45 (7 days) MPO^+^ cells (per field) in vehicle-treated mice at the times indicated. For CD3^+^ cells the quantification showed: 27.54 ± 2.72 (24 h); 14.41 ± 1.24 (72 h); 14.89 ± 1.65 (7 days) CD3^+^ cells (per field) in VCE-004.8-treated group vs 41.67 ± 2.20 (24 h); 34.02 ± 2.31 (72 h); 40.05 ± 4.70 (7 days) CD3^+^ cells (per field) in vehicle-treated mice at the times indicated. Furthermore, macrophage recruitment measured in the pericontusional area by F4/80 staining was strongly reduced in VCE-004.8-treated mice group compared to the vehicle-treated group (Additional file 1: Figure S4C). Quantification of immunolabeled images with F4/80 marker is showed in Fig. 10C (14.64 ± 1.89 (24 h); 12.31 ± 0.71 (72 h); 24.06 ± 2.21 (7 days) F4/80 staining (Area %) in VCE-004.8-treated group vs 29.98 ± 5.84 (24 h); 25.05 ± 3.15 (72 h); 40.65 ± 2.90 (7 days) F4/80 staining (Area %) in vehicle-treated mice at the times indicated). These results are fully consistent with the previous results showing the effect of VCE-004.8 in the maintenance of BBB integrity.

### Effects of VCE-004.8 on inflammatory markers and apoptosis in the brain of TBI mice

**Fig. 11:**
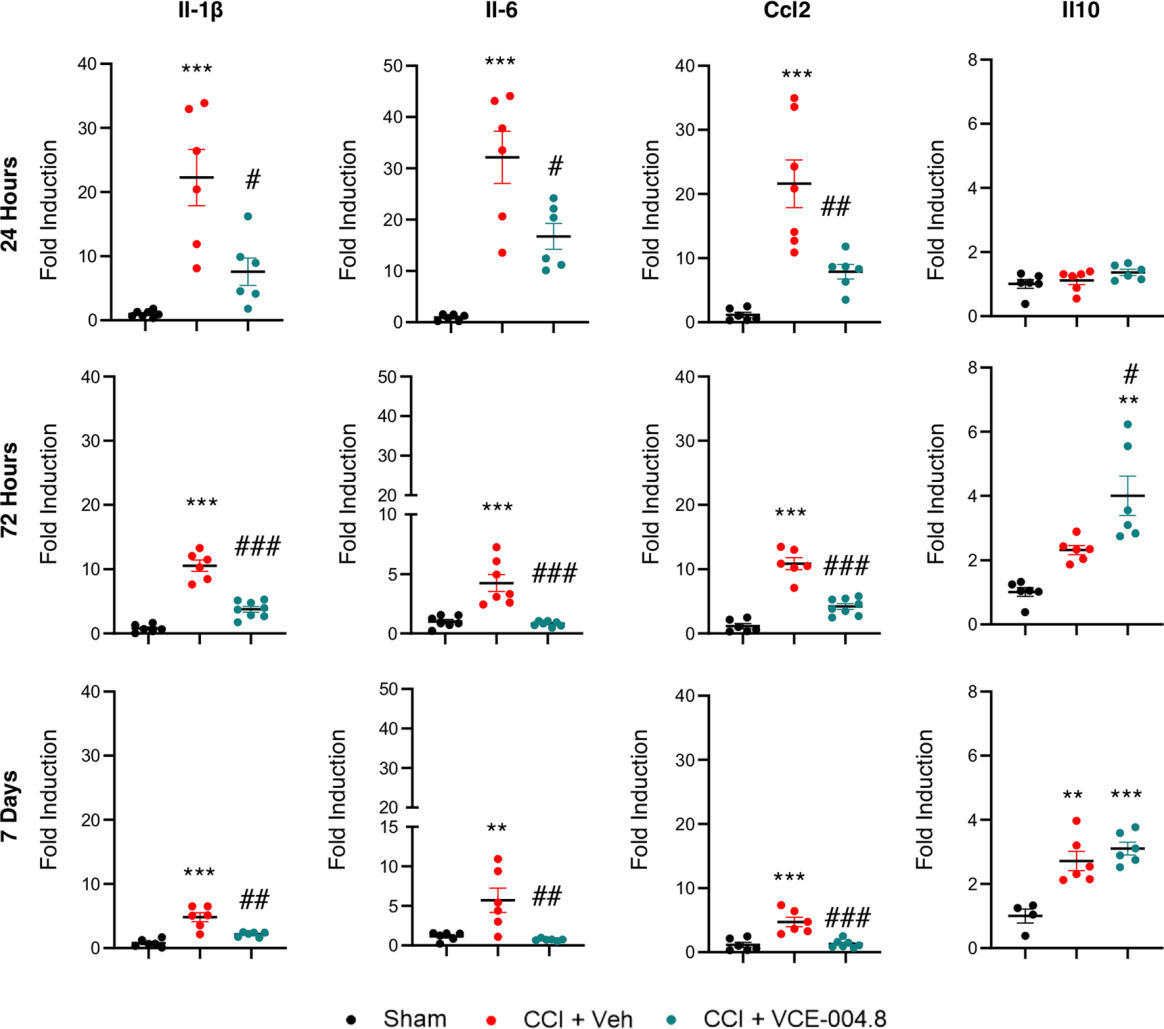
VCE-004.8 attenuates the expression of inflammatory cytokines in TBI. The mRNA expression for inflammatory markers in the pericontusional cortex (Il-1β, Il-6, Ccl2 and Il10) was quantified by qPCR and normalized versus Ppia at 24 h, 72 h and 7 days. Data are represented as mean ± SEM, and significance was determined by one-way ANOVA followed by the Tukey’s post hoc test. Il-1β (24 h: *p* = 0.0001; ***p < 0.001, 72 h: *p* < 0.0001; ***p < 0.001, 7 days: *p* = 0.0002; ***p < 0.001 CCI + Vehicle vs Sham, 24 h: *p* = 0.0113; #*p* < 0.05, 72 h: *p* = 0.0001; ###*p* < 0.001, 7 days: *p* = 0.0011; ##*p* < 0.01 CCI + VCE-004.8 vs CCI + Vehicle). Il-6 (24 h: *p* = 0.0004; ***p < 0.001, 72 h: *p* = 0.0001; ***p < 0.001, 7 days: *p* = 0.0040; **p < 0.01 CCI + Vehicle vs Sham, 24 h: *p* = 0.0211; #*p* < 0.05, 72 h: *p* < 0.0001; ###*p* < 0.001, 7 days: *p* = 0.0018; ##*p* < 0.01 CCI + VCE-004.8 vs CCI + Vehicle). Ccl2 (24 h and 72 h: *p* < 0.0001; ***p < 0.001, 7 days: *p* = 0.0008; ***p < 0.001 CCI + Vehicle vs Sham, 24 h: *p* = 0.0058; ##*p* < 0.01, 72 h: p < 0.0001; ###*p* < 0.001, 7 days: *p* = 0.0009; ###*p* < 0.001 CCI + VCE-004.8 vs CCI + Vehicle). Il-10 (72 h: *p* = 0.0018; **p < 0.01 CCI + VCE-004.8 vs Sham, p = 0.0449; #*p* < 0.05 CCI + VCE-004.8 vs CCI + Vehicle, 7 days: *p* = 0.0013; **p < 0.01 CCI + Vehicle vs Sham, *p* = 0.0004; ***p < 0.001 CCI + VCE-004.8 vs Sham)

**Fig. 12:**
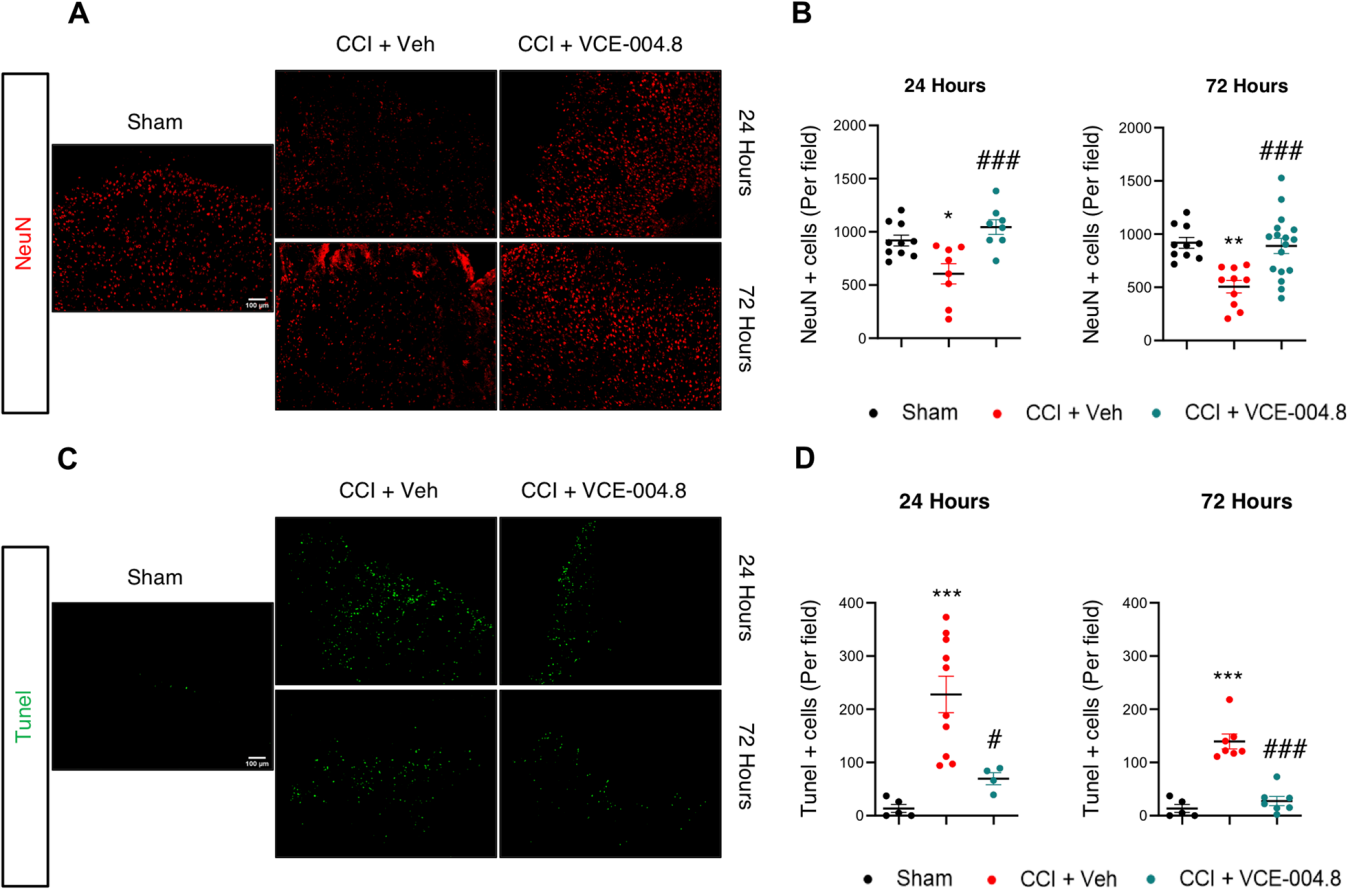
VCE-004.8 reduces apoptosis and neuronal death induced by TBI. **A**, **C** Representative confocal images showing NeuN (red) and Tunel (green) expression in pericontusional cortex at 24 h and 72 h after the brain injury. Scale bars equivalent to 100 µm (**B**–**D**). **B**, **D** The quantification of each marker is shown as mean ± SEM, and significance was determined by one-way ANOVA followed by the Tukey’s post hoc test. **B** (24 h: p = 0.0119; *p < 0.05, 72 h: *p* = 0.0014; **p < 0.01 CCI + Vehicle vs Sham, 24 h: *p* = 0.0010; ##*p* < 0.01, 72 h: *p* = 0.0009; ###*p* < 0.001 CCI + VCE-004.8 vs CCI + Vehicle). **D** (24 h: *p* = 0.0006; ***p < 0.001, 72 h: *p* < 0.0001; ***p < 0.001 CCI + Vehicle vs Sham, 24 h: *p* = 0.0133; #*p* < 0.05, 72 h: *p* < 0.0001; ###*p* < 0.001 CCI + VCE-004.8 vs CCI + Vehicle)

To further study the effect of VCE-004.8 on the expression of inflammatory mediators in the pericontusional area we determined the mRNA expression of several pro-inflammatory markers. As shown in Fig. 11, VCE-004.8 treatment decreased the expression of pro-inflammatory cytokines and chemokines such as Il-1β (7.66 ± 1.77 (24 h); 3.77 ± 0.44 (72 h); 2.15 ± 0.15 (7 days) fold induction in VCE-004.8-treated group vs 22.90 ± 4.50 (24 h); 10.39 ± 1.37 (72 h); 4.57 ± 0.50 (7 days) fold induction in vehicle-treated mice at the times studied), Il-6 (15.24 ± 3.47 (24 h); 0.81 ± 0.08 (72 h); 0.73 ± 0.05 (7 days) fold induction in VCE-004.8-treated group vs 33.88 ± 6.79 (24 h); 4.25 ± 0.71 (72 h); 5.93 ± 1.74 (7 days) in vehicle-treated mice at the times indicated), and Ccl2 (7.77 ± 0.73 (24 h); 4.20 ± 0.44 (72 h); 1.30 ± 0.23 (7 days) fold induction in VCE-004.8-treated group vs 22.17 ± 3.64 (24 h); 10.89 ± 1.14 (72 h); 4.71 ± 0.90 (7 days) in vehicle-treated mice at the times indicated) and enhanced the expression of the anti-inflammatory cytokine IL-10 (4.00 ± 0.61 (72 h); 3.10 ± 0,19 (7 days) fold induction in VCE-004.8-treated group vs 1.00 ± 0.21 in Sham group at both times; 4.00 ± 0.61 fold induction in VCE-004.8-treated mice vs 2.31 ± 0.17 in vehicle-treated mice at 72 h). Several studies demonstrate that acute neuronal damage appears after traumatic brain injury as a consequence of the inflammatory process. In fact, it has been described that the strongest labeling of injured cells in the cortex occurs acutely within the first 3 days post-injury [27]. Since VCE-004.8 improved neuroinflammation and BBB integrity after TBI, we expected that VCE-004.8 treatment could also reduce neuronal loss after brain injury. As depicted in Fig. 12A and B the expression of NeuN^+^ cells was significantly reduced in the cortex around the lesion in sham mice and this reduced expression was prevented by VCE-004.8 treatment after 24 and 72 h post-TBI (606.4 ± 95.18 (24 h); 505.4 ± 58.13 (72 h) NeuN^+^ cells (per field) in vehicle-treated mice vs 918.5 ± 50.18 in Sham group at both times; 1044 ± 69.17 (24 h); 889.1 ± 72.21 (72 h) NeuN^+^ cells (per field) in VCE-004.8-treated mice vs 606.4 ± 95.18 (24 h); 504.4 ± 58.13 (72 h) in vehicle-treated mice at the times indicated). In addition, TUNEL assay was used to detect cell apoptosis in the pericontusional area. We show in Fig. 12C, D that the proportion of apoptotic cells in the vehicle-treated mice group was considerably higher than in VCE-004.8-treated mice group (227.8 ± 34.34 (24 h); 139.6 ± 13.97 (72 h) Tunel^+^ cells (per field) in vehicle-treated mice vs 13.60 ± 7.56 in Sham group at both times; 69.50 ± 11.30 (24 h); 27.57 ± 8.67 (72 h) Tunel^+^ cells (per field) in VCE-004.8-treated mice vs 227.8 ± 34.34 (24 h); 139.6 ± 13.97 (72 h) in vehicle-treated mice at the times indicated).

## Discussion

TBI is the major cause of long-term disability among children and young adults in the developed world [28]. Current literature suggests around 70 million individuals worldwide are estimated to suffer TBI each year [29] and an estimated cost of more than $400 billion annually on a global scale [30, 31]. One of the main complications in treating TBI is the heterogeneity of its mechanism from a point of view of pathologic and pathogenic triggers. Although the primary injury caused by the mechanical force is considered untreatable, the subsequent secondary damage offers a period for treatment and has therefore attracted more attention in recent years.

In this study, we demonstrated that VCE-004.8 stabilizes HIF-1α by modifying PHD2 activity. VCE-004.8 is not an enzymatic inhibitor of PHDs and through MS/MS studies and immunoblots using a phospho-specific Ser-125-PHD2 antibody, we have demonstrated that the compound induces a clear reduction on the Ser-125 phosphorylation levels. In this regard, a previous study has shown that PHD2 phosphorylation at Ser-125 is modulated or controlled by the coordinated action of the kinase P70S6K and the phosphatase PP2A/B55α [12]. Although genetic silencing of B55α resulted in a clear reduction of the stabilization of HIF-1α, we cannot discard that VCE-004.8 also targets P70S6K. Moreover, it is also possible that VCE-004.8 is targeting the phosphorylation of endogenous inhibitors regulating PP2A/B55α such as Arspp19 and α-endosulfine, which are regulated by the Greatwall kinase [32]. Experiments are in progress to investigate those possibilities.

It has been shown that an increase in the expression levels of HIF-1α exerts a neuroprotective activity in cerebral ischemia, hypoxia injury and TBI [33–35]. In situations of hypoxia and cerebral ischemia, there is a self-protective mechanism mediated by an increase in the expression of HIF-1α that allows the brain to increase the tolerance to hypoxia through transcriptional and post-transcriptional mechanisms. For instance, pharmacological inhibition of PHDs maintains mitochondrial function and protects neuronal cells from glutamate-induced oxytosis [36]. In addition, HIF-1α stabilization induces the expression of genes such as erythropoietin (Epo) or vascular endothelial growth factor (Vegf) that have been closely linked to neuroprotection [33, 37–39]. Interestingly, mild hypoxia induced by hypoxic chambers or by hypoximimetic compounds such as PDHi has been shown to provide neuroprotective activity in vivo [40]. Consequently, the increase in expression of HIF-1α after TBI could be acting as a neuroprotective agent, thus improving the prognosis of patients.

One of the main benefits of brain angiogenesis is the increase of the blood flow and oxygen supplied to the brain tissue, providing an important neurovascular niche for neuronal restoration and functional improvement after TBI [41, 42]. In this regard, it has been described that the role of PP2A/B55α in vascular remodeling is promoting endothelial cells survival in a HIF-dependent manner [18]. We have previously demonstrated that VCE-004.8 upregulates the expression of HIF-dependent genes such as Epo and Vegf, induces angiogenesis in vitro and enhances migration of oligodendrocytes [19]. Now we show that VCE-004.8 significantly increased the formation of functional vessels (CD31/α-SMA) after 7 days of treatment in a Matrigel plug assay. Furthermore, the expression of CD31^+^/Ki67^+^ cells showed that the presence of new vascular endothelial cells was higher in the VCE-004.8-treated mice group than in mice which only received vehicle in the TBI model. Angiogenesis and neurogenesis play an important role in mediating functional recovery after experimental stroke and TBI [42, 43] [44–46]. Our findings suggest that VCE-004.8-mediated angiogenesis could improve the blood and oxygen supply to the brain, preventing infiltration of peripheral immune cells and reducing the level of apoptosis and neuronal death in the pericontusional area.

Clinical data show that TBI-associated damage is accompanied by vascular endothelial dysfunction, where both arterioles and capillaries located in the cerebral cortex are severely damaged leading to BBB breakdown [6, 47]. Vascular endothelium-associated damages include the loss of tight junctions (TJs) proteins and astrocyte activation among others [6, 48]. TJs are represented by transmembrane proteins occludins, claudins and the accessory proteins zonula occludens (ZO) family (ZO-1 and ZO-2), whose function is to establish a physical barrier against paracellular diffusion [49, 50] [51, 52]. Claudin-5 and ZO-1 are key proteins to maintain the integrity and proper functioning of the BBB [53]. Importantly, it has been described that small molecules that enhance or restore claudin-5 expression improve transendothelial electrical resistance and reduce the BBB permeability [54]. Our results showed that claudin-5 and ZO-1 decreased after TBI and the treatment with VCE-004.8 restored the expression of both TJs proteins that paralleled with the amelioration of prevented brain edema after the traumatism. Preliminary data indicate that the therapeutic window last for 3 h after CCI, but a detailed identification of this window needs to be explored. Although the mechanism of action remains to be determined, a relationship between PP2A inhibition and claudin-5 expression in brain endothelial cells has been described [55]. Furthermore, it has been described, although not directly, that the PP2A activator FTY720 improved the BBB disruption after TBI, preventing brain edema by up-regulating claudin-5 [56]. However, FTY720 does not activate the HIF pathway and probably other PP2A isoforms are also involved in BBB protection.

Several hallmarks associated with neuroinflammation are a deficient functioning of the BBB, infiltration of the brain parenchyma by leukocytes and uncontrolled activation of glial cells [57, 58]. Glial cells include oligodendrocytes, responsible for generating and maintaining the myelin sheaths of neurons, astrocytes, which provide neuronal support and control the functioning of the BBB at biochemical and structural levels, and microglia, resident myeloid cells whose function is to collaborate in the maintenance of the CNS homeostasis as well as to mediate inflammatory responses to an injury or infection. Activated microglia are responsible for the production of several inflammatory factors closely related to neurotoxicity. In this sense, TNF-α produced by microglia induces inflammatory responses promoting the secondary brain injury [59]. In TBI and other neuroinflammatory pathologies such as multiple sclerosis monocytes and microglia adopt a pro-inflammatory state called the M1 phenotype [60–62]. On the other hand, astrocytes also play an important role controlling the BBB function and participating in CNS regeneration after brain injury [63, 64]. Once the inflammatory response continues, the reactive astrocytes rapidly proliferate and migrate to the damaged site to form glial scar that isolate the injured area, providing a favorable situation for neuron survival and maintenance of BBB integrity [65, 66]. However, it has been described that the glial scar has a negative impact on axonal regeneration and recovery of synaptic connectivity [67, 68]. There is an abundance of evidence demonstrating at the preclinical levels that agonists either for peroxisome proliferator-activated receptor-γ (PPARγ) or cannabinoid type 2 receptor (CB_2_R) have potent anti-inflammatory and neuroprotective activities. For instance, glitazones, a class of PPARγ ligand full agonists, show anti-inflammatory and neuroprotective activities in animal models of TBI [69–72]. Also, the expression of CB_2_R in the brain is mainly restricted to activated microglia and specific ligand agonists for this receptor show efficacy in neuroinflammatory diseases including TBI [73–75]. Thus, it is important to highlight that VCE-004.8 is also a dual PPARγ/CB_2_ agonist with potent anti-inflammatory activities in different murine inflammatory models [19, 20, 76–78]. Moreover, VCE-004.8 dampens IL-17-induced M1 polarization and inhibits LPS-induced prostaglandin E2 synthesis in macrophages and microglia. These effects could be mediated through PPARγ and CB_2_ signaling [78]. Our results show that VCE-004.8 inhibits the expression of proinflammatory cytokines Il-1β, Il-6 and Ccl2 after TBI could be explained by the immunomodulatory activity of VCE-004.8 through a combined effect mediated by PPARγ/CB_2_ activation, but further experiments are required to investigate this possibility.

During the last few years there has been an increase in the search and development of PP2A-activating drugs (PADs). They are being investigated not only as possible treatments for cancer, but also for inflammatory and neurodegenerative diseases including tauopathies such as Alzheimer’s disease (AD), [79, 80]. Indeed, it has been described that PP2A activity is reduced by about 50% in AD-affected CNS associated with several mechanisms of PP2A dysfunction [81, 82]. This link between TBI and the development of AD and other neurodegenerative diseases paved the way for the development of novel therapeutic avenues providing benefits in the short and long term after TBI. To achieve this goal the clinical development of small molecules with specific multipronged attributes such as VCE-004.8 and other cannabidiol derivatives are of special interest.

## Conclusions

TBI is a very complex physiopathological condition and different pathogenic mechanisms are involved in the short- and long-term clinical outcomes. Drug candidates preventing BBB disruption, peripheral immune cell infiltration and endowed with anti-inflammatory and neuroprotective activities are of great interest for the management of acute and secondary brain injury in TBI. Herein we provide evidence that the treatment with VCE-004.8, a dual PPARγ/CB_2_R agonist that also activates B55α/PHD2/HIF pathway, ameliorated BBB disruption and neuroinflammation, provided neuroprotection and improved neurological function after TBI. Therefore, identifying new strategies to alleviate neuroinflammatory responses and to protect from vascular endothelial cells damage might be crucial for the treatment of TBI and perhaps other neurological diseases cursing with neuroinflammation and BBB disruption.

## Supplementary Information

**Additional file 1. **Supplementary Figures.

## Data Availability

Data sharing is not applicable to this article as no datasets were generated or analyzed during the current study.
