# Supplementary material for: A cannabidiol aminoquinone derivative activates the PP2A/B55α/HIF pathway and shows protective effects in a murine model of traumatic brain injury
Source: J Neuroinflammation. 2022 Jul 9;19:177. doi: 10.1186/s12974-022-02540-9 (PMC9270745; doi:10.1186/s12974-022-02540-9)
Supplement: Supplementary file 1 — Additional file 1. Supplementary Figures. [file 12974_2022_2540_MOESM1_ESM.pdf]

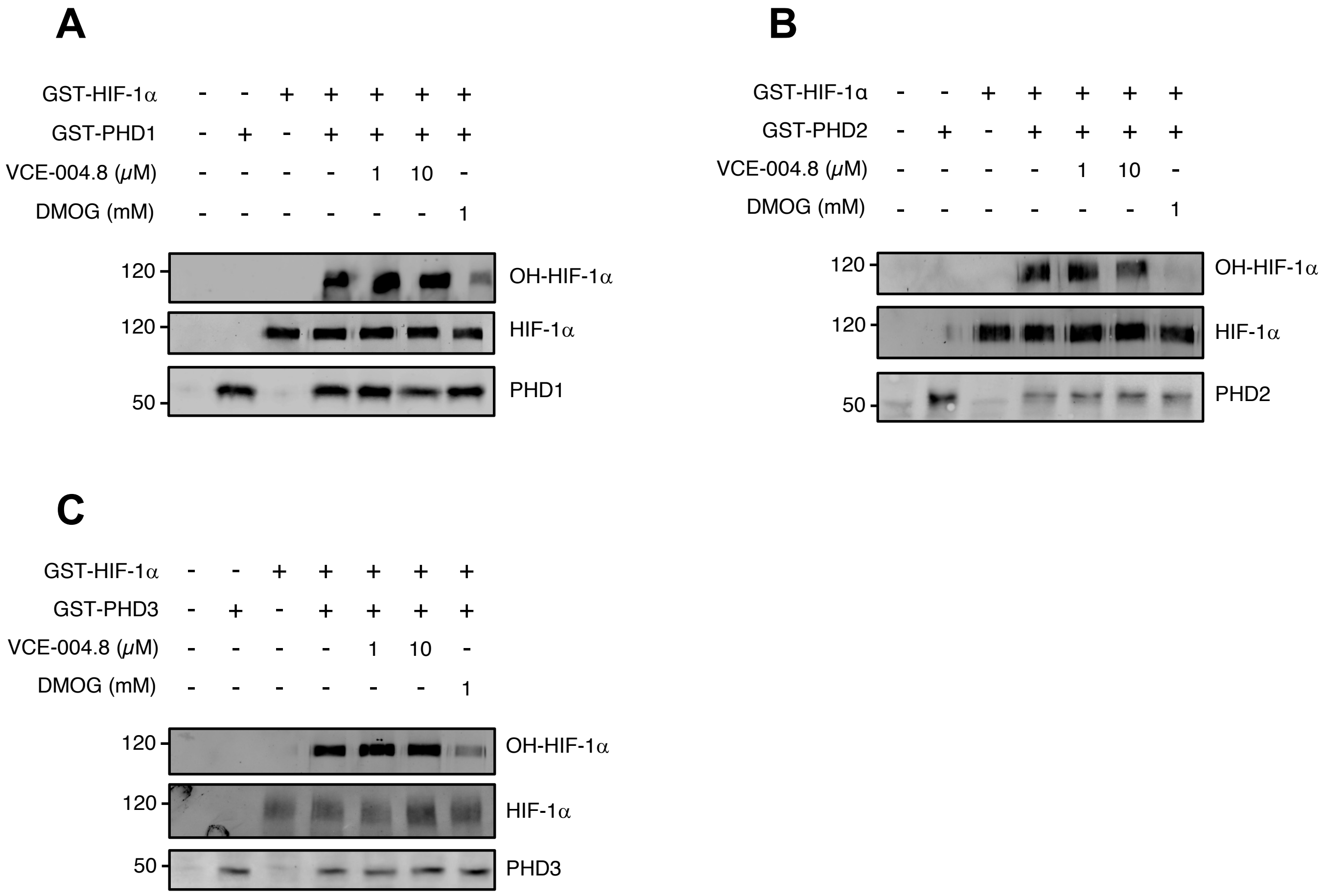

Figure Supplementary 1. Effect of VCE-004.8 on HIF-1α hydroxylation mediated by PHDs. (A-C) HIF-1α *in vitro* hydroxylation reaction was carried out employing recombinant human HIF-1α and PHDs proteins, obtained according to standard protocols in BL21 bacterial cells and purified using Glutathione-Sepharose 4B (GE Healthcare). Proteins were incubated in the reaction buffer 50 mM Tris/HCl (pH 7.5), 1 mM DTT, 50 μM FeSO4, 5 mM ascorbate, and 200 μM oxoglutarate for 1 hour at 30°C, in the presence of different concentrations of VCE-004.8 and using DMOG as positive control. The prolyl hydroxylation reaction was stopped by adding Laemmli sample buffer and hydroxylation levels as well as total HIF-1α and PHDs signals were analyzed by western blot.

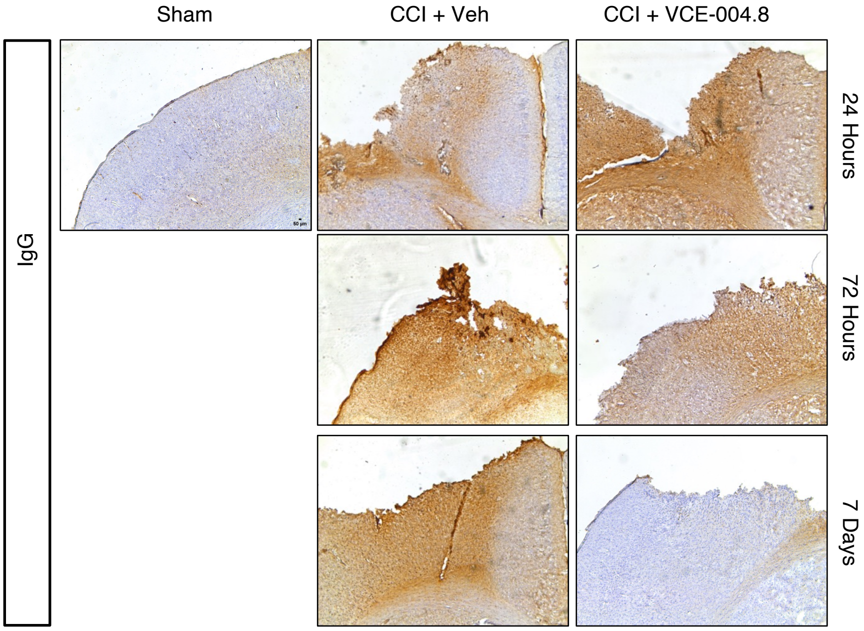

Figure Supplementary 2. Effect of VCE-004.8 on peripheral IgG infiltration into the brain. Representative immunohistochemically images of peripheral IgG infiltration in the pericontusional cortex after the brain injury. Scale bar equivalent to 50 μm.

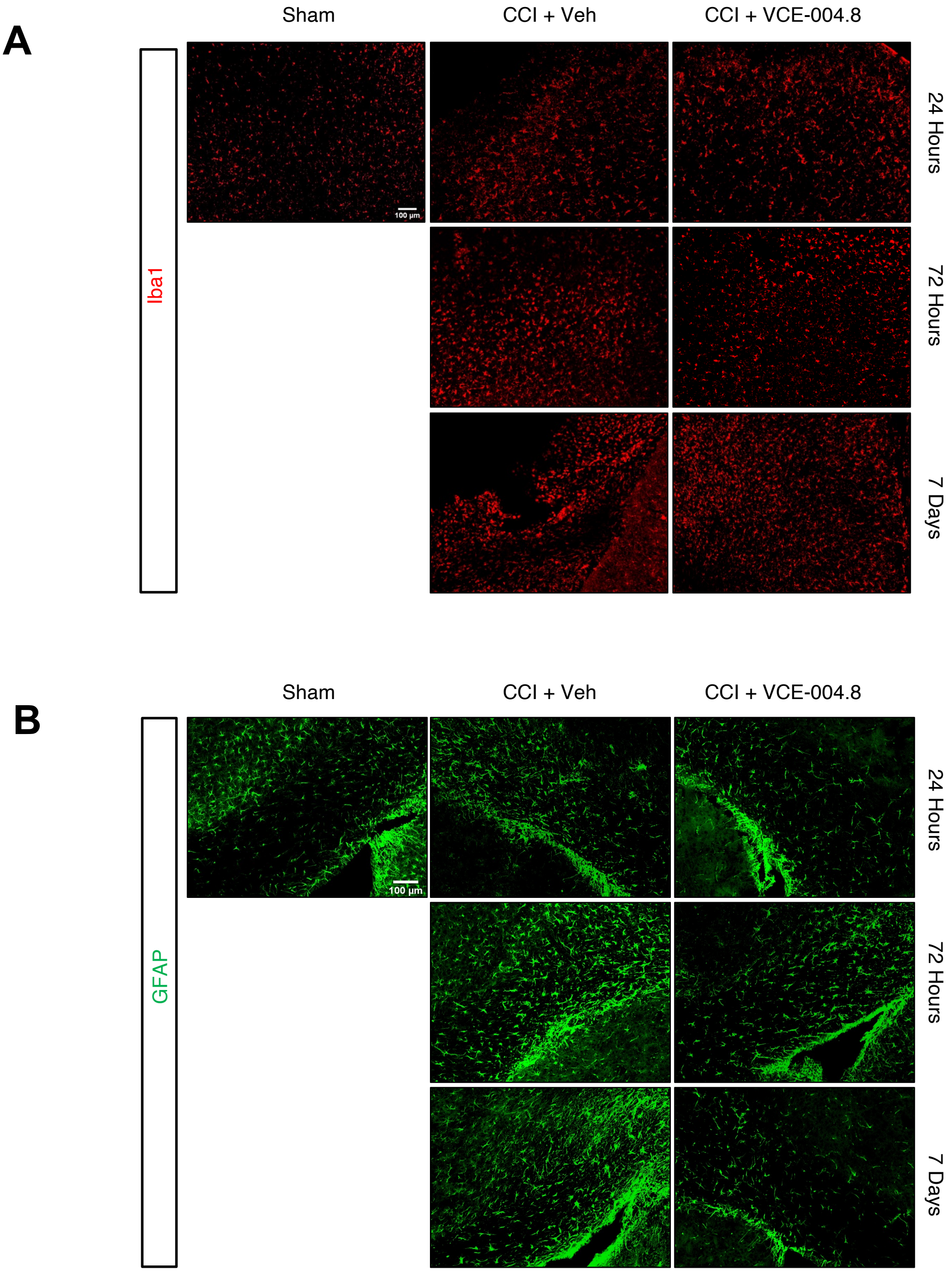

Figure Supplementary 3. Effect of VCE-004.8 on glia reactivation after TBI. (A) Representative confocal images showing Iba1$^+$ cells in pericontusional cortex at 24 hours, 72 hours and 7 days after the brain injury. (B) Representative confocal images showing GFAP$^+$ cells in Corpus Callosum at 24 hours, 72 hours and 7 Days post CCI. Scale bars equivalent to 100 µm.

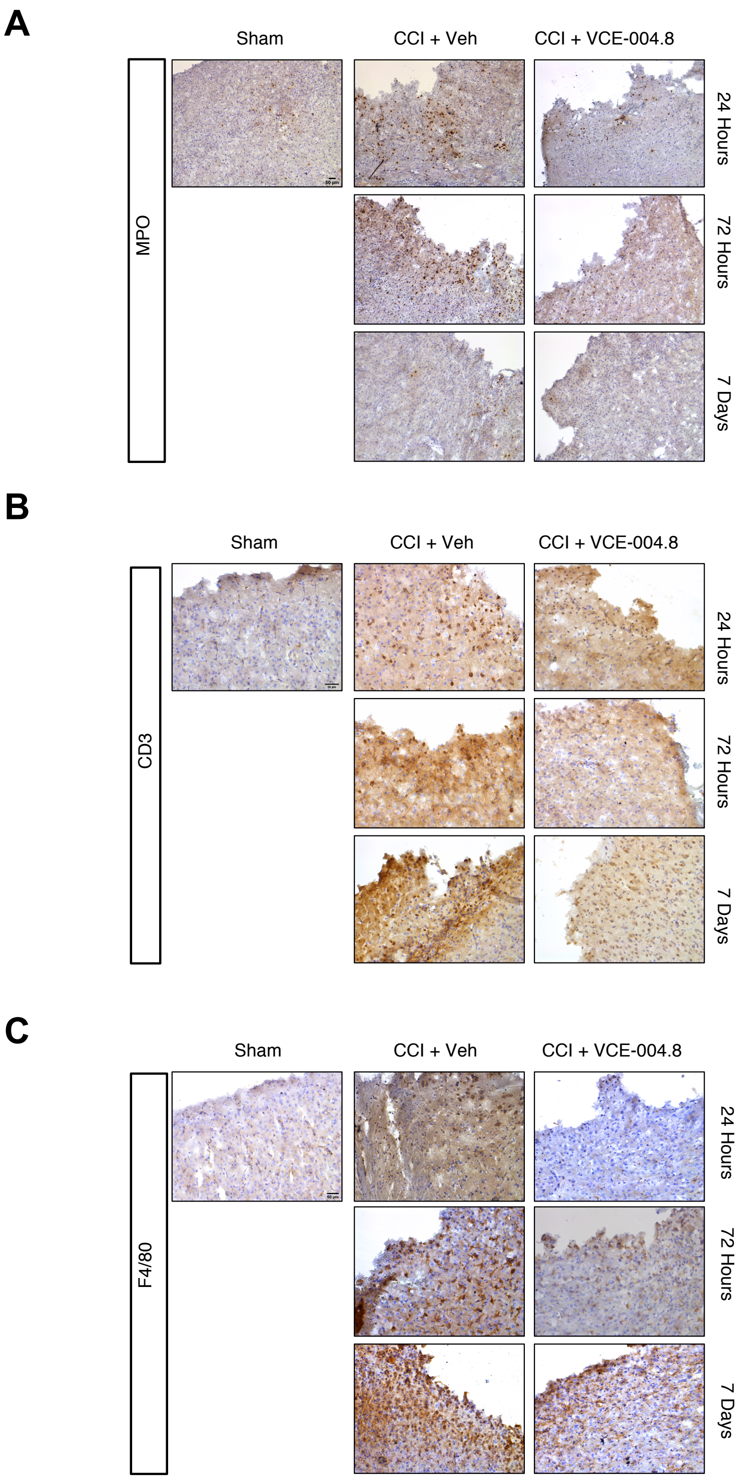

Figure Supplementary 4. Effect of VCE-004.8 on peripheral immune cells infiltration after TBI. Representative immunohistochemically images of neutrophils (MPO, A), T cells (CD3, B) and macrophages (F4/80, C) in the pericontusional cortex after the brain injury. Scale bars equivalent to 50 µm.

A

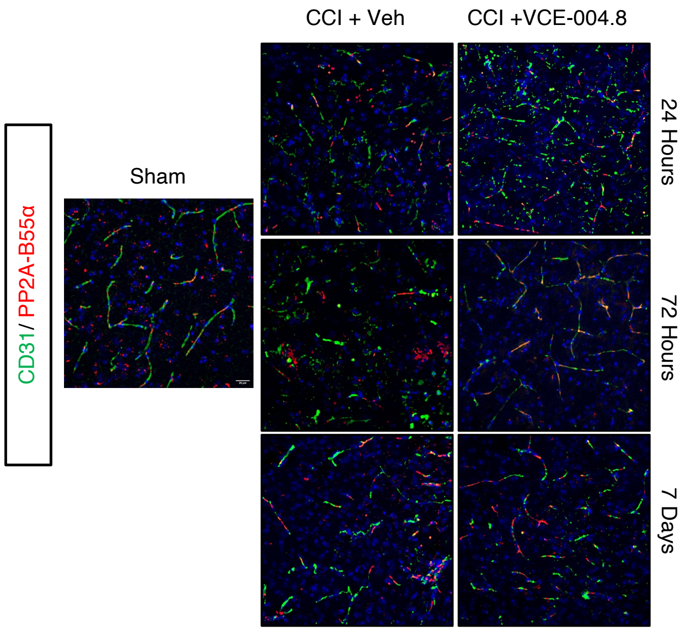

B

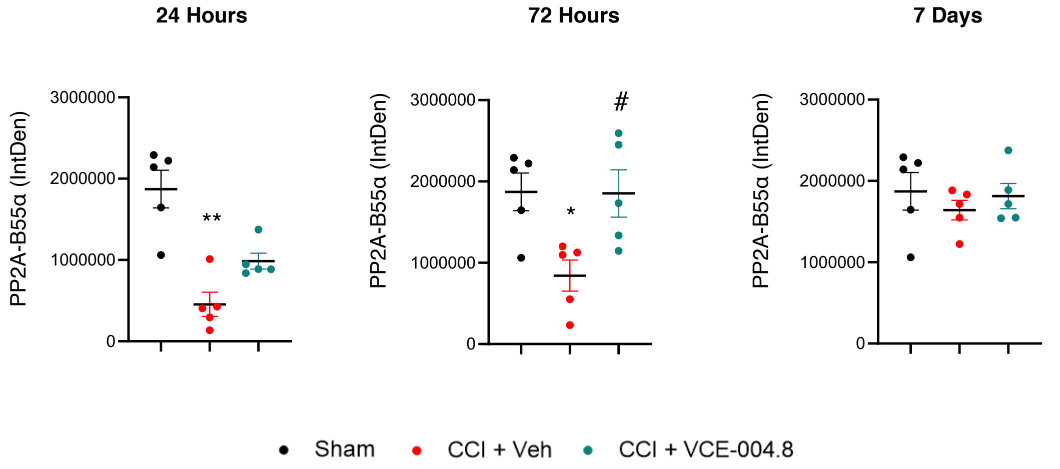

Figure Supplementary 5. Effect of VCE-004.8 on PP2A-B55α expression after TBI. (A) Representative confocal images for CD31 and PP2A-B55α expression in the pericontusional cortex at 24 hours, 72 hours and 7 days after the brain injury. Scale bar equivalent to 25 µm. (B) The quantification of PP2A-B55α (Integrated Density) is represented as mean ± SEM, and significance was determined by one-way ANOVA followed by the Tukey's post hoc test or Kruskas-Wallis post hoc test. 24 hours: p= 0.0044; *p<0.05 CCI + Vehicle vs Sham, 72 hours: p<0.0268; *p<0.05 CCI + Vehicle vs Sham; p= 0.0297; # 0.05 CCI + VCE-004.8 vs CCI + Vehicle.

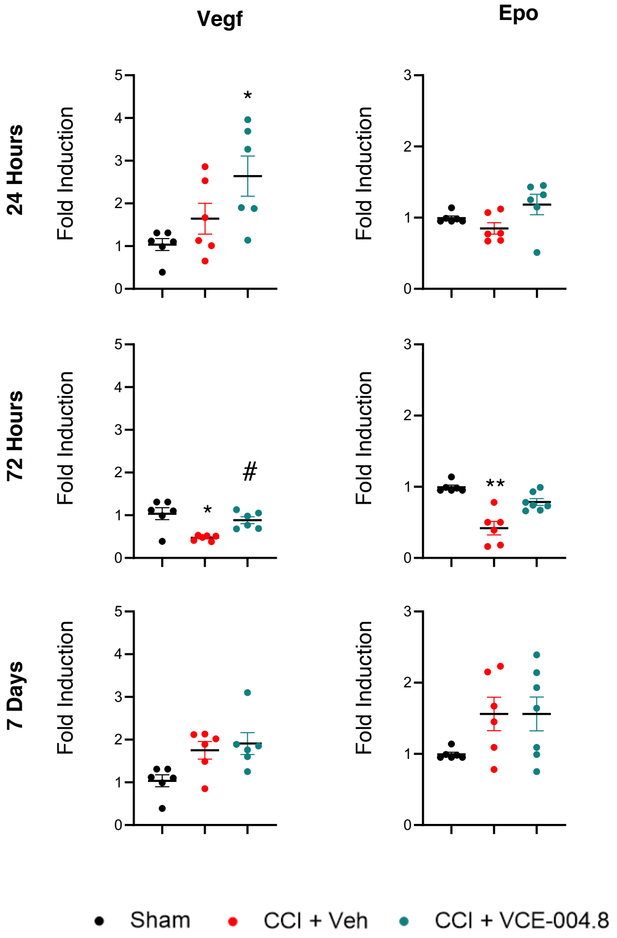

Figure Supplementary 6. Effect of VCE-004.8 on the expression of HIF-1α-dependent genes. The mRNA expression for Vegf and Epo in the pericontusional cortex was quantified by qPCR and normalized versus Ppia at 24 hours, 72 hours and 7 days. Data are represented as mean ± SEM, and significance was determined by one-way ANOVA followed by the Tukey's post hoc test or Kruskal-Wallis post hoc test. Vegf (24 hours: $p = 0.0333$; *$p<0.05$ CCI + VCE-004.8 vs Sham, 72 hours: $p = 0.0171$ *$p<0.05$ CCI + Vehicle vs Sham, $p = 0.04046$; #$p<0.05$ CCI + VCE-004.8 vs CCI + Vehicle). Epo (72 hours: $p = 0.0065$; **$p<0.01$ CCI + Vehicle vs Sham).
